# TRPA1 activation and Hsp90 inhibition synergistically downregulate macrophage activation and inflammatory responses in vitro

**DOI:** 10.1186/s12865-023-00549-0

**Published:** 2023-06-30

**Authors:** Anukrishna Radhakrishnan, Tathagata Mukherjee, Chandan Mahish, P Sanjai Kumar, Chandan Goswami, Subhasis Chattopadhyay

**Affiliations:** 1 National Institute of Science Education and Research, an Off-campus Centre (OCC) of Homi Bhabha National Institute, Bhubaneswar, Odisha 752050 India; 2 Institute of Life Sciences, Nalco Nagar Rd, NALCO Square, NALCO Nagar, Chandrasekharpur, Bhubaneswar, Odisha 751023 India

**Keywords:** Macrophages, Pro-inflammatory responses, TRPA1, 17-AAG, Apoptosis, Ca^2+^

## Abstract

**Background:**

Transient receptor potential ankyrin 1 (TRPA1) channels are known to be actively involved in various pathophysiological conditions, including neuronal inflammation, neuropathic pain, and various immunological responses. Heat shock protein 90 (Hsp90), a cytoplasmic molecular chaperone, is well-reported for various cellular and physiological processes. Hsp90 inhibition by various molecules has garnered importance for its therapeutic significance in the downregulation of inflammation and are proposed as anti-cancer drugs. However, the possible role of TRPA1 in the Hsp90-associated modulation of immune responses remains scanty.

**Results:**

Here, we have investigated the role of TRPA1 in regulating the anti-inflammatory effect of Hsp90 inhibition via 17-(allylamino)-17-demethoxygeldanamycin (17-AAG) in lipopolysaccharide (LPS) or phorbol 12-myristate 13-acetate (PMA) stimulation in RAW 264.7, a mouse macrophage cell lines and PMA differentiated THP-1, a human monocytic cell line similar to macrophages. Activation of TRPA1 with Allyl isothiocyanate (AITC) is observed to execute an anti-inflammatory role via augmenting Hsp90 inhibition-mediated anti-inflammatory responses towards LPS or PMA stimulation in macrophages, whereas inhibition of TRPA1 by 1,2,3,6-Tetrahydro-1,3-dimethyl-N-[4-(1-methylethyl)phenyl]-2,6-dioxo-7 H-purine-7-acetamide,2-(1,3-Dimethyl-2,6-dioxo-1,2,3,6-tetrahydro-7 H-purin-7-yl)-N-(4-isopropylphenyl)acetamide (HC-030031) downregulates these developments. LPS or PMA-induced macrophage activation was found to be regulated by TRPA1. The same was confirmed by studying the levels of activation markers (major histocompatibility complex II (MHCII), cluster of differentiation (CD) 80 (CD80), and CD86, pro-inflammatory cytokines (tumor necrosis factor (TNF) and interleukin 6 (IL-6)), NO (nitric oxide) production, differential expression of mitogen-activated protein kinase (MAPK) signaling pathways (p-p38 MAPK, phospho-extracellular signal-regulated kinase 1/2 (p-ERK 1/2), and phosphor-stress-activated protein kinase/c-Jun N-terminal kinase (p-SAPK/JNK)), and induction of apoptosis. Additionally, TRPA1 has been found to be an important contributor to intracellular calcium levels toward Hsp90 inhibition in LPS or PMA-stimulated macrophages.

**Conclusion:**

This study indicates a significant role of TRPA1 in Hsp90 inhibition-mediated anti-inflammatory developments in LPS or PMA-stimulated macrophages. Activation of TRPA1 and inhibition of Hsp90 has synergistic roles towards regulating inflammatory responses associated with macrophages. The role of TRPA1 in Hsp90 inhibition-mediated modulation of macrophage responses may provide insights towards designing future novel therapeutic approaches to regulate various inflammatory responses.

**Supplementary Information:**

The online version contains supplementary material available at 10.1186/s12865-023-00549-0.

## Background

The transient receptor potential (TRP) superfamily integrates 30 closely related non-selective cationic channels, distributed into seven subfamilies and two groups based on their sequence similarity and cellular functions [1, 2]. Subfamilies of TRP channels are named TRPC (Canonical), TRPV (Vanilloid), TRPM (Melastatin), TRPA (Ankyrin), TRPML (Mucolipin), TRPP (Polycystin), and TRPN (NOMPC). TRP channels are found in both excitable and non-excitable vertebrate cells and some non-vertebrate cells, contributing to essential cellular functions [1, 3–6]. TRP channels are pivotal in various cellular processes, including cell division, migration, differentiation, stress responses, and apoptosis [7–9].

TRPA1, the only member of the mammalian TRPA family, is characterized by 14 ankyrin repeats in its N-terminus domain [10]. TRPA1 is required for various immune cells such as T lymphocytes and monocyte/macrophages in regulating their activation, migration, and secretion of different immune molecules [11–15]. In a recent study, it has been reported an important role of TRPA1 in regulating T cell activation and associated responses [16]. TRPA1 is essential in multiple inflammatory and anti-inflammatory functions in different model systems, including tissue injury, inflammatory models, and pain modalities. Recently, it has been reported that in inflammatory models such as acute kidney injury, atopic dermatitis model, and experimental colitis model, the TRPA1 expression levels were significantly elevated at the site of injury or inflammation [17–19]. Further, inflammatory reactions such as pro-inflammatory cytokine release and mast cell infiltration were impaired considerably upon genetic or pharmacological ablation of TRPA1 [20]. TRPA1 modulates pain induction and aggravates injury-induced inflammation. The protective role of TRPA1 is evident in various inflammatory immune responses, including corneal wound healing and mechanical or cold allodynia in chronic post-ischemia pain [21–23]. Similarly, TRPA1 is associated with lipopolysaccharide (LPS) induced inflammatory responses, including lung inflammation, neurogenic inflammation, and Osteoarthritic Fibroblast-Like Synoviocytes [24–26]. Activation of TRPA1 alleviates the LPS-induced nitric oxide (NO) production in peritoneal macrophages [27]. Like other TRP superfamily members, TRPA1 is associated with various cellular proteins essential for cell survival, including Hsp90, Hsp27, and Hsp70 [28–31].

Hsp90, a cytoplasmic molecular chaperone, is associated with the stabilization and maturation of cellular client proteins and helps in cell fate decisions, including cell cycle, signal transduction, growth regulation, and cell death [32, 33]. Hsp90 is essential for various pathophysiological conditions like cancer, viral infections, and autoimmune disorders [34–39]. Additionally, Hsp90 has been reported to effectively modulate different immune responses by regulating various client proteins involved in innate and adaptive immune responses [40]. Hsp90 inhibition by various pharmacological inhibitors has proven effective in (alleviating) a wide range of inflammatory responses, including macrophage-mediated pro-inflammatory responses, interleukin-1 receptor-associated kinase, Raf-1, mitogen-activated protein kinase kinase, and Src family kinase p56lck activation [41–46]. 17-AAG, a derivative of geldanamycin, is one of the selective inhibitors of Hsp90 and has been reported to actively block various innate immune responses in vitro and in vivo models. 17-AAG administration has been shown to suppress TLR4-mediated pro-inflammatory cytokine production via blockade of the signaling cascade during LPS-induced autoimmune uveitis in rats [47]. Furthermore, 17-AAG inhibits TLR4 stimulation in vitro and alleviates disease incidence and severity in myelin oligodendrocyte glycoprotein-peptide-induced experimental autoimmune encephalomyelitis [48]. These reports suggest the immense therapeutic potential of Hsp90 inhibitors in autoimmune and pro-inflammatory diseases. Although Hsp90 and TRPA1 have been well studied for their immune modulatory effect, the possible association of these proteins and the functional regulation of their effects has not been addressed yet. Accordingly, here we have investigated the association of Hsp90 inhibition-mediated anti-inflammatory effects and the possible contextual involvement of TRPA1-mediated immune regulation, if any. In this study, we have explored the role of TRPA1 in regulating pro-inflammatory responses in Hsp90-inhibited macrophages when subjected to LPS or PMA stimulation. Additionally, we have also studied the regulation of MAPK signalings, apoptosis, intracellular calcium status, and associated immune responses via 17-AAG and TRPA1 agonist Allyl isothiocyanate (AITC) in macrophages in LPS or phorbol 12-myristate 13-acetate (PMA) stimulation.

## Results

### TRPA1 is upregulated in Hsp90-inhibited and LPS-stimulated macrophages

**Fig. 1:**
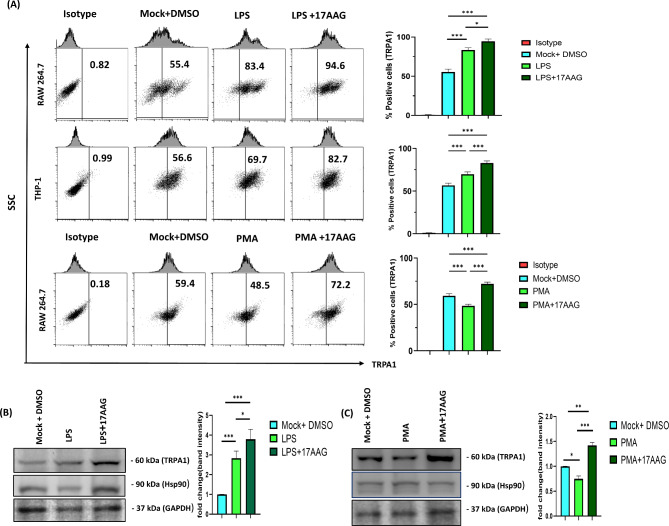
TRPA1 is upregulated in Hsp90-inhibited and LPS- or PMA-stimulated monocytes/macrophages. Cells were treated with either LPS 500 ng/ml (both RAW 264.7 and THP-1 macrophages) or PMA 100 ng/ml (only in RAW 264.7) alone or together with 17-AAG. (**A**) FC dot-plot and bar graphs depicting the percentage of positive cells for TRPA1 in mock, LPS/PMA, and 17-AAG + LPS/PMA. Western blot analysis and corresponding bar graphs of TRPA1 expression in RAW 264.7 cells stimulated with LPS (500 ng/ml) (**B**), PMA (100 ng/ml) (**C**), and 17-AAG + LPS/PMA. The blot figures were cropped to omit other conditions. The data represent the mean ± SD of three independent experiments. One-way ANOVA has been performed to find statistical significance among groups. Differences between groups with a p-value < 0.05 were considered statistically significant ( *, p < 0.05; **, p < 0.01; ***, p < 0.001)

TRPA1 is associated with a wide range of cellular and pathophysiological conditions [18, 21, 49, 50]. It has a protective role in macrophage-mediated inflammation in several inflammatory diseases [17, 23, 51–55]. The Hsp90 inhibitor used in the study is 17-AAG, which is accredited as a potential anti-inflammatory agent during LPS stimulation in macrophages via blockade of TLR4 signaling pathways [47, 48]. The working concentration of 17-AAG in RAW 264.7 cells was taken as 0.5 µM as more than 90% of the cells were viable at that concentration (Supplementary Fig. 2A) [56]. To investigate a possible association between TRPA1 and Hsp90-inhibition mediated impairment of inflammation in macrophages, RAW 264.7 cells or THP-1 macrophages were treated with either LPS/PMA or 17-AAG or together. The working concentration of LPS, PMA, and 17-AAG used were 500 ng/mL, 100 ng/mL, and 0.5 µM, respectively. These cells were then harvested, stained, and analyzed to check TRPA1 expression levels via flow cytometry (FC). The TRPA1 antibodies used are specific for mouse TRPA1 proteins, and the specificity was tested using blocking peptides (data not shown). The percentage of cells positive for TRPA1 was observed to be increased significantly in LPS-stimulated RAW 264.7 cells (83.4 ± 1.73%) as compared to resting RAW (mock) 264.7 cells (55.4 ± 3.73%) (Fig. 1A). Further, in macrophages treated with both 17-AAG and LPS, the TRPA1 levels were augmented (94.6 ± 1.67%). Similarly, it was found that the percentage of cells positive for TRPA1 decreased significantly in PMA-stimulated RAW 264.7 cells (48.5 ± 1.74%) as compared to resting RAW 264.7 cells (59.5 ± 2.19%). Furthermore, in macrophages treated with both 17-AAG and PMA, the TRPA1 levels were higher (72.2 ± 1.90%) (Fig. 1A). The samples from each condition were assessed for TRPA1 protein quantification via Western blot. The highest band intensity for TRPA1 was obtained in LPS/PMA stimulated, and 17-AAG treated conditions (Fig. 1B and C). The THP-1 macrophages also followed a similar trend with reaching maximum TRPA1 levels in Hsp90-inhibited and LPS-stimulated macrophages (82.8 ± 2.53%), trailed by LPS-stimulated macrophages (69.7 ± 2.72%) and resting macrophages (56.6 ± 2.61%). These results suggest that the TRPA1 levels are modulated during LPS or PMA stimulation in a dose- and time-dependent manner. Furthermore, increased TRPA1 expression was observed after the administration of both 17-AAG and LPS as compared to the mock (untreated) macrophages in a dose-independent and reversible manner (Supplementary Fig. 1). The results indicate a possible modulation of TRPA1 expression in Hsp90-inhibited macrophages during LPS or PMA stimulation.

### TRPA1 regulates the activation of Hsp90-inhibited macrophages

To investigate whether the differential expression of TRPA1 in the above conditions has any functional implication, TRPA1 specific agonist (AITC) and TRPA1 antagonist (HC-030031) were used [57, 58]. The cytotoxicity levels of the TRPA1-specific modulators were assessed by trypan blue exclusion assay and 7-AAD staining via FC. RAW 264.7 cells were treated with different concentrations of TRPA1 modulators HC-030031 (TRPA1 inhibitor) (40 µM, 20 µM, 10 µM, 5 µM) and AITC (TRPA1 activator) (40 µM, 20 µM, 10 µM, 5 µM) in the presence of 17-AAG for 24 h. DMSO was used as solvent control. More than 95% of the cells were viable at 10 µM and 5 µM of HC-030031 in the presence of 0.5 µM of 17-AAG, and similar results were observed at 20 µM, 10 µM, and 5 µM of AITC in the presence of 0.5 µM of 17-AAG (Supplementary Fig. 2). Henceforth, 10 µM of AITC and 10 µM of HC-030031 were used for further experiments. It was also observed that these pharmacological modulators alone or in combination with 17-AAG have no significant effect on TRPA1 levels in the absence of any inflammatory stimulus (Supplementary Fig. 3).

**Fig. 2:**
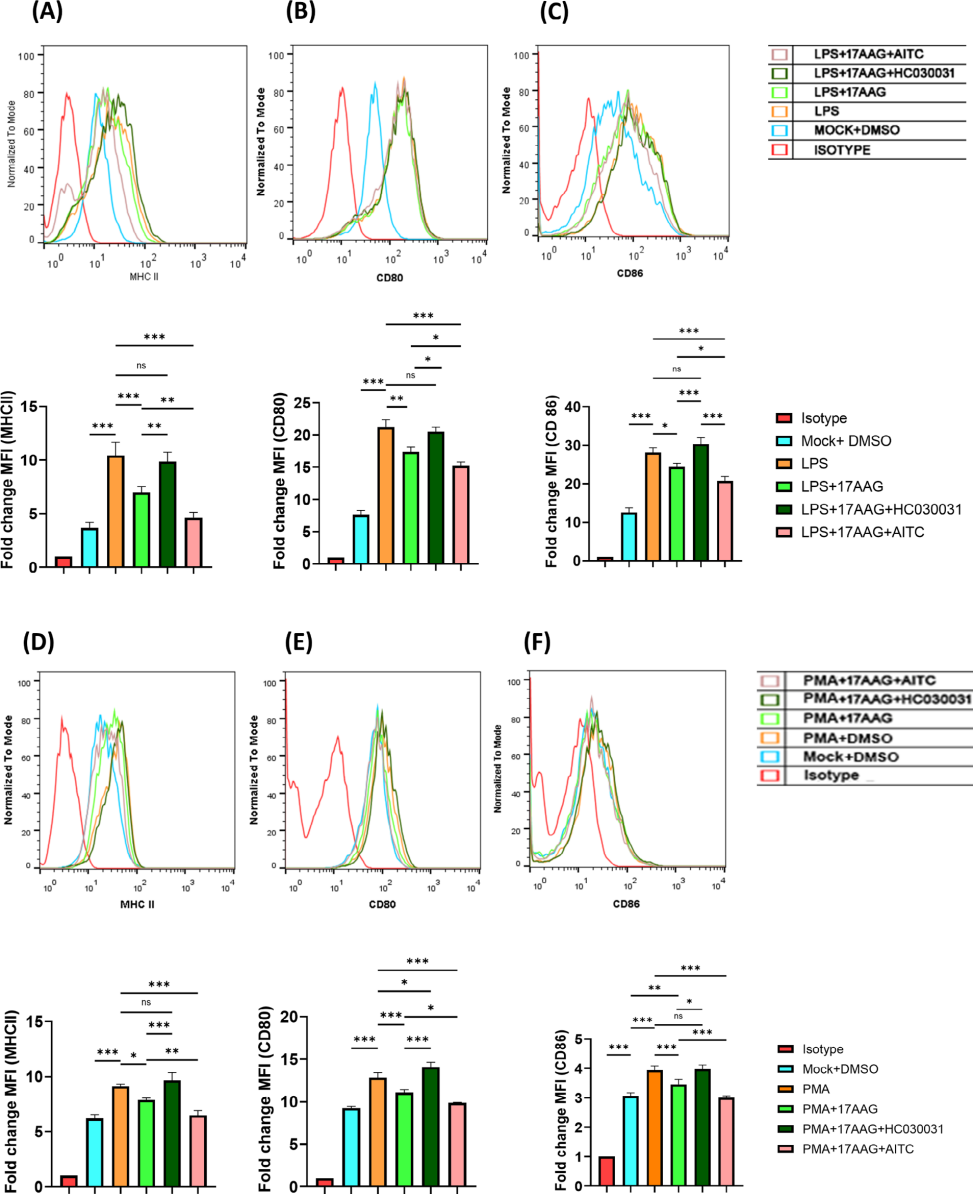
TRPA1 regulates the activation of macrophages in Hsp90-inhibited condition. RAW 264.7 cells were treated with different conditions of LPS/PMA, 17-AAG, HC-030031, or AITC and harvested at 12 h. FC histogram depicting fold change in MFI of MHCII (**A**, **D**), CD80 (**B**, **E**), and CD86 (**C**, **F**) of macrophages treated with LPS (500ng/ml, A-C) or PMA (100 ng/ml, D-F) along with their respective bar graphs. The data represent the mean ± SD of at least three independent experiments. One-way ANOVA has been performed to find statistical significance among groups. Differences between groups with a p-value less than 0.05 were considered statistically significant (ns, non-significant; *, p < 0.05; **, p < 0.01; ***, p < 0.001)

To determine whether TRPA1 has any role in regulating the activation of Hsp90-inhibited macrophages, cell surface expression of MHCII and CD80/86 were studied via FC; RAW 264.7 were stimulated with LPS or PMA in the presence of TRPA1 modulators and 17-AAG. The cells were harvested at 12 h post-stimulation, immunolabelled with MHCII, CD80, and CD86 antibodies, followed by their acquisition and analysis via FC (Fig. 2A-F). The expression levels of MHCII, CD80, and CD86 were represented in fold change compared to the isotype control. It was observed that inhibition of Hsp90 significantly decreases the expression of MHCII (6.99 ± 0.51), CD80 (17.4 ± 0.72), and CD86 (24.4 ± 0.94) as compared to the LPS stimulated cells (MHCII: 10.40 ± 1.23, CD80: 21.2 ± 1.14 and CD86: 28.1 ± 1.2). Furthermore, the pharmacological inhibition of TRPA1 with HC-030031 significantly downregulated the effect of Hsp90 inhibition (MHCII: 9.85 ± 0.87, CD80: 20.5 ± 0.68, and CD86: 30.3 ± 1.65) as compared to LPS + 17-AAG. Conversely, TRPA1 activation with AITC significantly enhanced the Hsp90-mediated downregulation of MHCII (4.63 ± 0.51), CD80 (15.2 ± 0.58), and CD86 (20.7 ± 1.09) as compared to LPS + 17-AAG (Fig. 2A and B, and Fig. 2C). Similarly, it was observed that Hsp90 inhibition has significantly downregulated the expression of MHCII (7.90 ± 0.17), CD80 (11.1 ± 0.32), and CD86 (3.46 ± 0.16) as compared to the control PMA-stimulated cells (MHCII: 9.13 ± 0.195, CD80: 12.8 ± 0.59 and CD86: 3.95 ± 0.14). Further, pharmacological inhibition of TRPA1 with HC-030031 significantly reduced the effect of Hsp90 inhibition (MHCII: 9.65 ± 0.73, CD80: 14.1 ± 0.55, and CD86: 3.98 ± 0.13) as compared to PMA + 17-AAG. Conversely, TRPA1 activation with AITC has significantly enhanced the Hsp90-mediated downregulation of MHCII (6.50 ± 0.424), CD80 (9.87 ± 0.06), and CD86 (3.01 ± 0.05) as compared to PMA + 17-AAG (Fig. 2D and E, and Fig. 2F). These results indicate an important role of TRPA1 in the suppression of activation markers of macrophages i.e., MHCII, CD80, and CD86 in Hsp90-inhibited conditions in the presence of LPS or PMA stimulation.

### TRPA1 impairs the nitric oxide (NO) production in Hsp90-inhibited macrophages

**Fig. 3:**
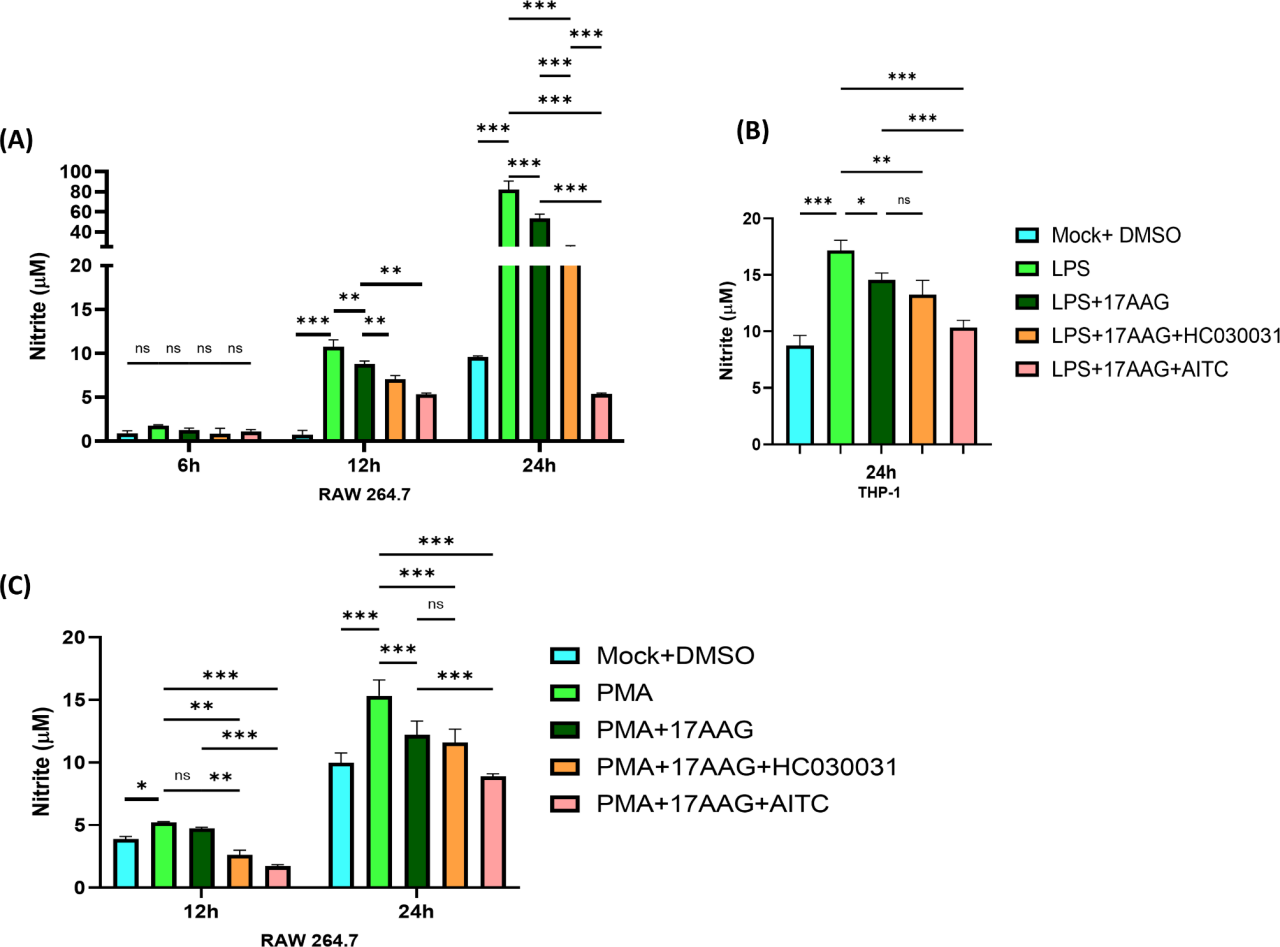
TRPA1 regulates the nitric oxide (NO) production in Hsp90-inhibited monocytes/macrophages. RAW 264.7 and THP-1 cells were treated with different conditions of LPS/PMA, 17-AAG, HC-030031, or AITC, and the supernatant was collected at 6 h,12 h, and 24 h. Bar graph depicting nitric oxide production from RAW 264.7 cells treated with LPS (500 ng/ml) (**A**) or PMA (100 ng/ml) (**C**) or THP-1 macrophages treated with LPS (500 ng/ml) (**B**). The data represent the mean ± SD of at least three independent experiments. One-way/two-way ANOVA was performed to find statistical significance among groups. Differences between groups with a p-value less than 0.05 were considered statistically significant (ns, non-significant; *, p < 0.05; **, p < 0.01; ***, p < 0.001)

Hsp90 is an active modulator of reactive nitrogen species (RNS) and reactive oxygen species (ROS) [59, 60]. To investigate the regulatory effect of TRPA1 in regulating the NO production by 17-AAG-mediated Hsp90 inhibited condition, RAW 264.7 and THP-1 macrophages were stimulated with LPS or PMA in the presence of TRPA1 modulators and 17-AAG. Griess assay was performed from the cell supernatants to assess the nitrite, a breakdown product of NO [61]. Upon LPS stimulation, it was observed that the nitrite production was upregulated at 24 h (78.7 ± 8.39 µM) as compared to the untreated cells (9.59 ± 0.831 µM). Further, upon Hsp90 inhibition with 17-AAG, the nitrite production was significantly downregulated (53.6 ± 2.42 µM). Surprisingly, in the presence of either HC-030031 (21.58 ± 1.42) or AITC (5.38 ± 0.667 µM), the NO levels decreased significantly (Fig. 3A). This trend was observed at 12 h, while no significant changes in NO production were observed at 6 h post-stimulation. A similar scenario was observed with THP-1 macrophages stimulated with LPS. Activation of TRPA1 along with 17-AAG (10.3 ± 0.6 µM) significantly impaired the NO production compared to the LPS 17.2 ± 0.891 µM) and LPS + 17-AAG (14.6 ± 0.97 µM) at 24 h conditions (Fig. 3B). Additionally, a significant uprise in nitrite production was observed in macrophages treated with PMA at 12 and 24 h. Furthermore, TRPA1 activation diminished the nitrite production in 17-AAG treated and PMA stimulated macrophages successfully compared to the PMA and PMA + 17-AAG controls at 12 and 24 h. Surprisingly, no significant changes were observed with HC-030031 + 17-AAG conditions compared to 17-AAG control in PMA-stimulated macrophages (Fig. 3C). These results indicate that TRPA1 activation augments the downregulation of NO production via Hsp90 inhibition in LPS/PMA-stimulated macrophages.

### TRPA1 enhances the Hsp90 inhibition-mediated downregulation of pro-inflammatory cytokine production in LPS or PMA-stimulated macrophages

**Fig. 4:**
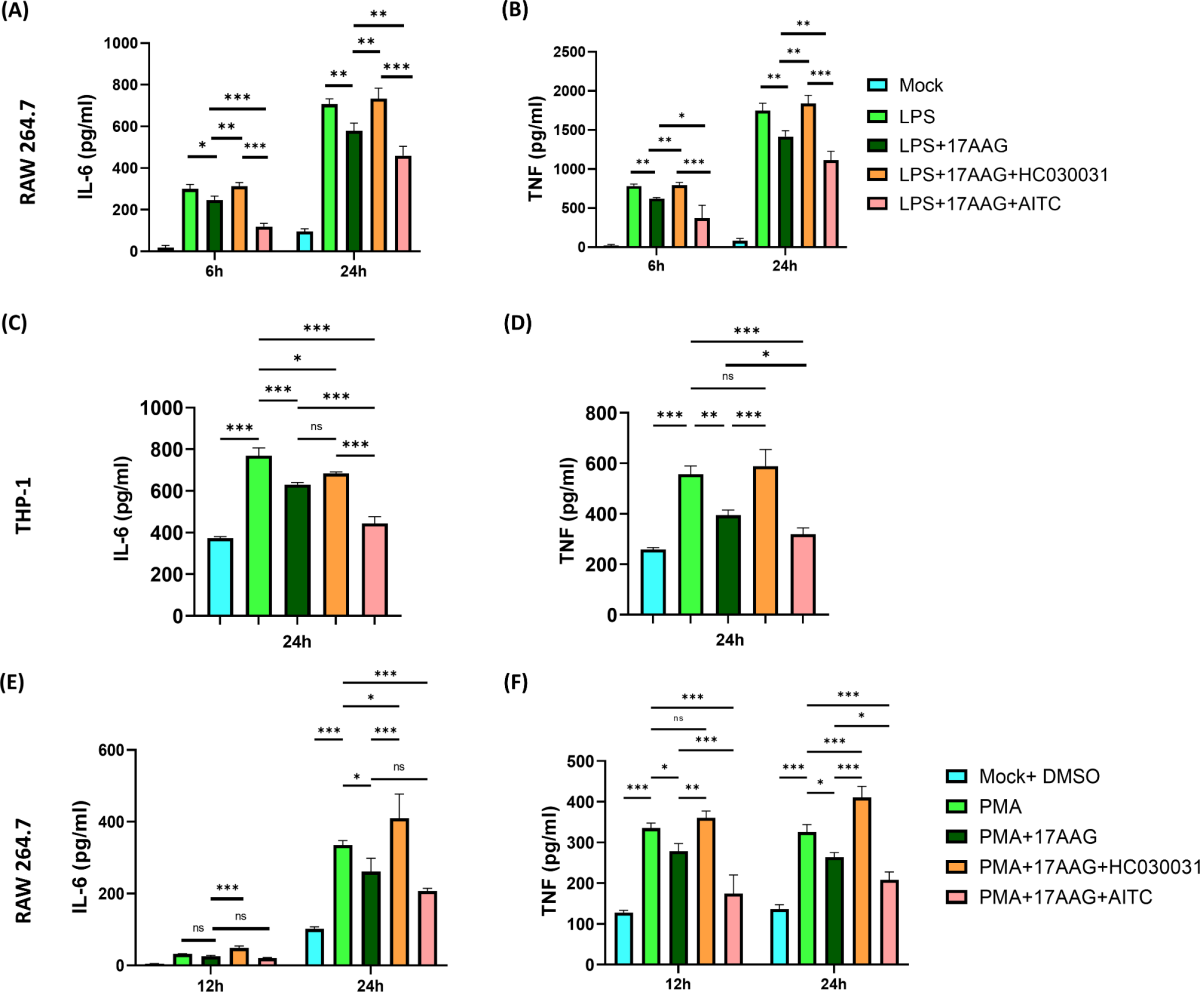
TRPA1 regulates the pro-inflammatory cytokine production in Hsp90-inhibited monocytes/macrophages. RAW 264.7 or THP-1 cells were subjected to different conditions of LPS/PMA, 17-AAG, HC-030031, or AITC, and the supernatant was collected at 6 and 24 h and assessed for cytokine profile. Bar graph representing IL-6 (**A**, **C**, **E**) and TNF (**B**, **D**, **F**) levels in RAW 264.7 cells stimulated with either LPS (500 ng/ml) (**A**) / PMA (100 ng/ml) (**C**) and THP-1 macrophages stimulated with LPS (500ng/ml) (**B**). The data represent the mean ± SD of three independent experiments. One-way/two-way ANOVA was performed to find statistical significance among groups. Differences between groups with a p-value less than 0.05 were considered statistically significant (ns, non-significant; *, p < 0.05; **, p < 0.01; ***, p < 0.001)

Hsp90 has been reported to be essential for pro-inflammatory cytokine production from macrophages [62]. To investigate the regulatory effect of TRPA1 in regulating the pro-inflammatory cytokine production by 17-AAG-mediated Hsp90 inhibited condition, RAW 264.7 cells were subjected to LPS or PMA stimulation under differential conditions of TRPA1 modulation and 17-AAG treatment. The culture supernatant was assessed for TNF and IL-6 cytokine release profiles. In 17-AAG-mediated Hsp90 inhibited and LPS- or PMA-stimulated macrophages, the TNF and IL-6 levels were reduced significantly at 6 and 24 h post-LPS-stimulation compared to only LPS or only PMA controls. Further, the inhibition of TRPA1 by HC-030031 has increased and restored the pro-inflammatory cytokine production in Hsp90-inhibited and LPS-stimulated macrophages, nullifying the effect of 17-AAG as the TNF and IL-6 production of LPS + 17-AAG + HC-030031 or PMA + 17-AAG + HC-030031 samples were comparable to only LPS control. Conversely, activation of TRPA1 in the LPS + 17-AAG + AITC or PMA + 17-AAG + AITC conditions alleviated the pro-inflammatory cytokine production compared to Hsp90-inhibited and LPS- or PMA-stimulated macrophages (Fig. 4). The TNF and IL-6 production were significantly downregulated compared to LPS or PMA, LPS + 17-AAG, and PMA + 17-AAG samples. Furthermore, AITC administration in LPS-stimulated macrophages could impair TNF and IL-6 production; however, HC-030031 could not significantly change LPS-stimulated macrophages (Supplementary Fig. 4). Similar results were observed with LPS-stimulated THP-1 macrophages at 24 h (Fig. 4B). These results indicate an important role of TRPA1 in the anti-inflammatory development effect induced by 17-AAG-mediated Hsp90 inhibition.

### TRPA1 modulates the Hsp90 inhibition-mediated downregulation of MAPK activation during LPS stimulation in macrophages

**Fig. 5:**
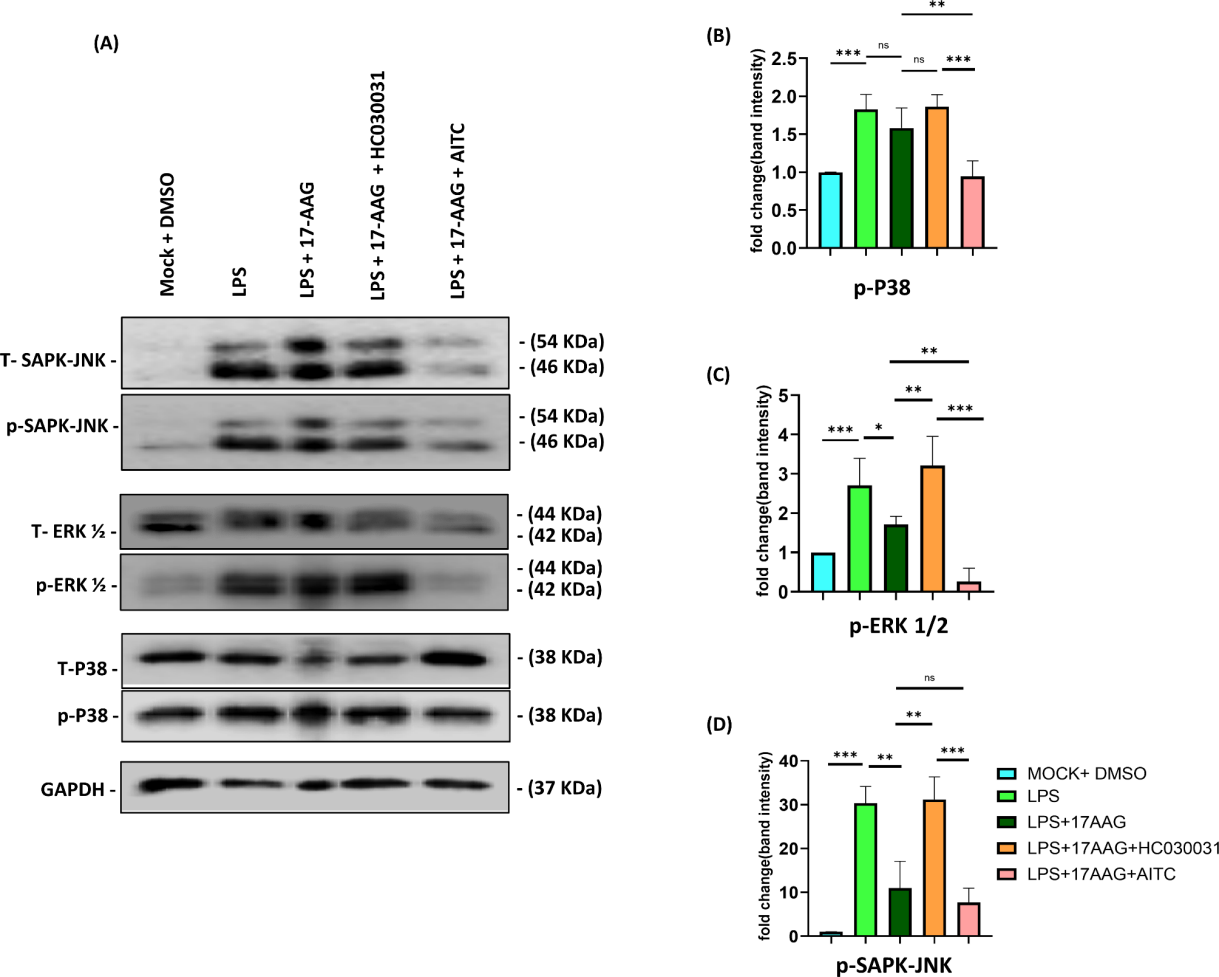
TRPA1 regulates the Hsp90 inhibition-mediated downregulation of MAPK signaling protein phosphorylation in LPS-stimulated monocytes/macrophages. RAW 264.7 cells were subjected to different conditions of LPS (500 ng/ml), 17-AAG, HC-030031, and AITC harvested at 15 min and assessed for intracellular signaling proteins p38-MAPK, ERK 1/2, SAPK-JNK and their respective phosphorylated proteins via western blot. (**A**) Western blot images from the samples represent p38-MAPK, ERK 1/2, SAPK-JNK, and their respective phosphorylated proteins. Bar graph representing the fold change in band intensity of phospho-proteins p-p38-MAPK (**B**), p-ERK 1/2 (**C**), and p-SAPK-JNK (**D**) normalized to the corresponding GAPDH controls. The data represent the mean ± SD of three independent experiments. Differences between groups with a p-value less than 0.05 were considered statistically significant (ns, non-significant; *, p < 0.05; **, p < 0.01; ***, p < 0.001)

Hsp90 has been well-attributed as a regulator of various signaling complexes of inflammation and associated responses [63]. Hsp90 and inhibitors of Hsp90 have been reported to be associated with the activation of ERK-MAPK signaling pathways and SAPK/JNK pathways in various immune models [64, 65]. To investigate the role of TRPA1 in Hsp90-mediated regulation in MAPK pathways, RAW 264.7 cells were subjected to LPS (500 ng/mL) stimulation under differential conditions of TRPA1 modulators and 17-AAG for 15 min. Samples were collected and assessed to quantify signaling proteins, p38-MAPK, ERK 1/2, SAPK/JNK, and their respective phosphorylated proteins via western blot. Interestingly, it was observed that Hsp90 inhibition via 17-AAG has significantly downregulated the LPS-induced p-p38-MAPK, p-ERK 1/2, and p-SAPK/JNK expression. Further, this development via 17-AAG was reversed with TRPA1 inhibition via HC-030031 treatment. The TRPA1 activation via AITC successfully diminished the expression of the proteins and signaling further compared to the respective 17-AAG + LPS and LPS conditions (Fig. 5). These results indicate that the TRPA1 is required for Hsp90 inhibition-mediated regulation of major MAPK signaling cascades.

### TRPA1 modulates Hsp90 inhibition-mediated apoptosis and inflammatory cytokine responses in activated macrophages

**Fig. 6:**
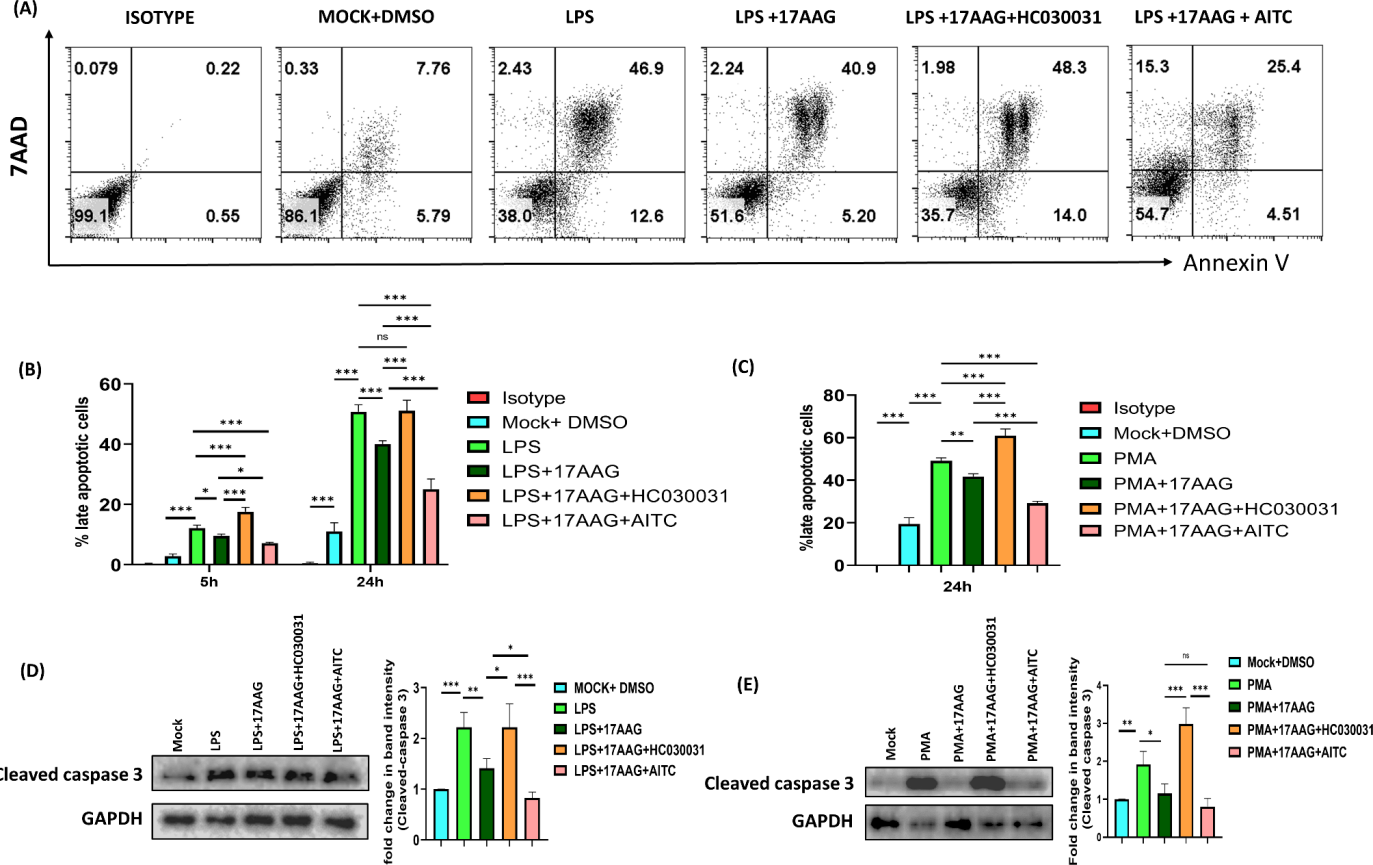
TRPA1 regulates apoptosis in Hsp90-inhibited and LPS/PMA-stimulated macrophages. RAW 264.7 cells were treated with different conditions of LPS (500 ng/ml)/PMA (100 ng/ml), 17-AAG, HC-030031, and AITC. Cells were harvested at 5 and 24 h. Heat-killed cells were used as a positive control. (**A**) FC dot plots representing the percentage of positive cells for Annexin V and 7-AAD at 24 h. Double-positive cells were considered either dead or late apoptotic. Representative bar graphs of sample stimulated with LPS (500 ng/ml) (**B**)/PMA (100 ng/ml) (**C**). (**D**, **E**) Western blot image and bar graph representing fold change in band intensity for cleaved caspase 3 (**D**, **E**) for the respective samples. The data represent the mean ± SD of three independent experiments. Differences between groups with a p-value less than 0.05 were considered statistically significant (ns, non-significant; *, p < 0.05; **, p < 0.01; ***, p < 0.001)

Hsp90 has been found to be involved in cell survival during various inflammatory and cancer models [38, 66]. Additionally, our group has reported that Hsp90 inhibition by 17-AAG downregulates the CHIKV-induced apoptosis in host macrophages [56]. To investigate the regulatory role of TRPA1 in Hsp90 inhibition-mediated developments in LPS-induced apoptosis of macrophages, if any, RAW 264.7 cells were incubated with differential conditions of TRPA1 modulators, 17-AAG, and LPS or PMA and assessed for cell death. We have performed cell-death analysis (Annexin V and 7-AAD staining) via FC. The cells were harvested at 5 and 24 h post-stimulation and assessed for apoptosis via Annexin V and 7-AAD staining followed by FC analysis (Fig. 6). We have found that cell death was increased significantly in LPS-stimulated macrophages at 5 h (12.2 ± 1.02%) and further augmented at 24 h (50.8 ± 2.30%) post-stimulation as compared to untreated cells at 5 h (2.85 ± 0.71%) and 24 h (11.1 ± 2.87%). As expected, it was significantly diminished with 17-AAG administration at 5 h (9.65 ± 0.55%) and 24 h (40.1 ± 1.05%) compared to LPS-treated cells. Interestingly, the TRPA1 inhibition via HC-030031 and 17-AAG has significantly upregulated at both 5 and 24 h (17.6 ± 1.40% and 51.1 ± 3.14%), the apoptosis compared to LPS + 17-AAG condition. Conversely, TRPA1 activation via AITC has dramatically diminished the apoptosis at 5 and 24 h (7.17 ± 0.36%) and (25.1 ± 3.36%). Activation of TRPA1 via AITC exhibited an anti-apoptotic effect in LPS-stimulated macrophages as it diminished cell death compared to LPS-stimulated cells. However, HC-030031 has not modulated the cell death in LPS-stimulated macrophages (Supplementary Fig. 4). Similar results were observed in PMA-induced apoptosis of macrophages. RAW 264.7 cells treated with PMA (49.1 ± 1.46%) were susceptible to apoptosis at 24 h compared to untreated cells (19.4 ± 2.90%). The highest percentage of apoptotic cells at 24 h was observed in samples treated with PMA + LPS + HC-030031 (60.9 ± 3.16%) and the lowest in cells treated with PMA + 17-AAG + AITC (29.2 ± 0.872%) compared to PMA + 17-AAG (41.7 ± 1.35%) and PMA controls (49.1 ± 1.46%). These samples were also assessed for caspase 3 protein levels via western blot (Fig. 6C). Band intensity levels were the lowest for cleaved caspase 3 in LPS/PMA + 17-AAG + HC-030031 samples compared to LPS/PMA, LPS/PMA + 17-AAG, and LPS/PMA + 17-AAG + HC-030031. Inhibition of TRPA1 with HC-030031 augmented the cleaved caspase 3 levels to the respective LPS/PMA samples, nullifying the effect of 17-AAG, indicating the important role of TRPA1 towards regulating the Hsp90-associated apoptosis of macrophages.

### TRPA1 is an important contributor to intracellular Ca^2+^-influx in Hsp90-inhibited and LPS-stimulated macrophages

**Fig. 7:**
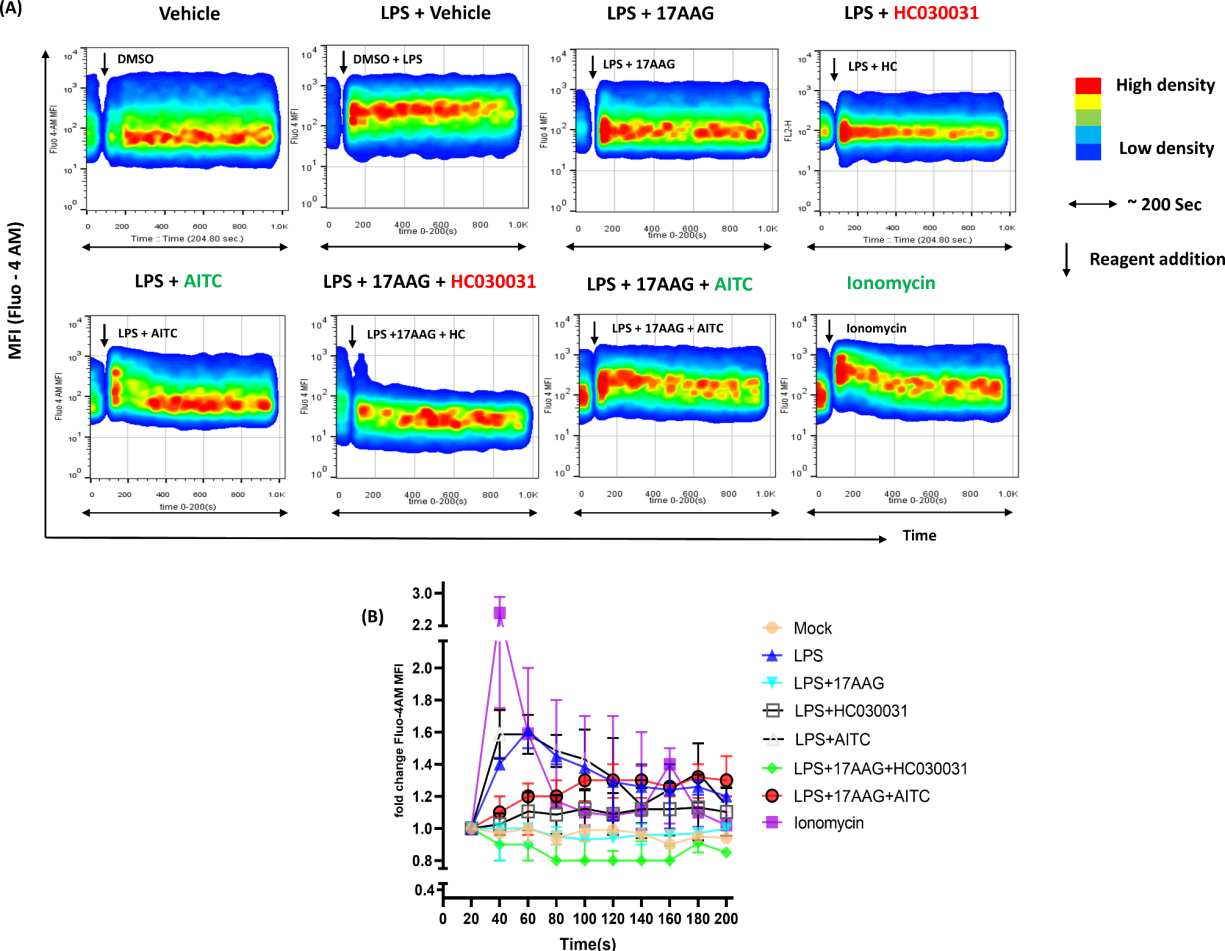
TRPA1 regulates intracellular calcium influx in LPS-stimulated and Hsp90-inhibited macrophages. RAW 264.7 cells were treated with Fluo-4 AM and assessed via FC for intracellular calcium levels upon combinatorial treatment with either ionomycin, vehicle (DMSO), vehicle + LPS, LPS + 17-AAG, LPS + 17-AAG + HC-030031, and LPS + 17-AAG + AITC. (**A**) Time-lapse kinetics of intracellular calcium influx. The X-axis of the flow cytometric plots represent approximately 200 s and ‘↓’ represents the addition of reagents/modulators to stimulate cells. (**B**) Representative line graph depicting fold changes in mean Fluo-4 intensity. The data represent the mean ± SD of three independent experiments.

Ca^2+^ currents via TRPA1 have been reported to modulate various immune responses and cell fate decisions [11, 12, 14, 17, 53, 67–69]. Studies have reported that intracellular Ca^2+^ increases after LPS stimulation [70]. To investigate the regulatory role of TRPA1 in intracellular Ca^2+^-influx in Hsp90-inhibited and LPS-stimulated macrophages, Ca^2+^-influx studies via FC were performed. RAW 264.7 cells were stained with Fluo-4 AM, and Ca^2+^-influx was analyzed via FC continuously for 200s. The mean value for every 20s interval was obtained, and two-way ANOVA was carried out for statistical analysis. The intracellular Ca^2+^ levels were compared before and after the addition of TRPA1 modulators, 17-AAG, and LPS in different conditions. Colorless RPMI media was used as a vehicle. Interestingly, we observed that the Ca^2+^ levels were augmented upon LPS stimulation in macrophages compared to mock or vehicle-treated cells, whereas 17-AAG administration could not evoke any changes in Ca^2+^-influx of its own (data not shown). Additionally, 17-AAG treatment along with LPS has significantly diminished the intracellular Ca^2+^ levels compared to the LPS-stimulation control. Similarly, HC-030031 administration reduced the elevated calcium levels during LPS stimulation, whereas the TRPA1 activation via AITC has upregulated it. HC-030031 treatment along with 17-AAG and LPS resulted in reduced Ca^2+^ levels compared to LPS only, LPS + 17-AAG, LPS + HC-030031. Additionally, the activation of TRPA1 via AITC along with 17-AAG and LPS resulted in elevated Ca^2+^-levels compared to LPS, or 17-AAG, LPS + 17-AAG, and LPS + AITC treated cells (Fig. 7). These results indicate that TRPA1 might be an important contributor to Ca^2+^-influx in Hsp90-inhibited and LPS-stimulated macrophages.

**Fig. 8:**
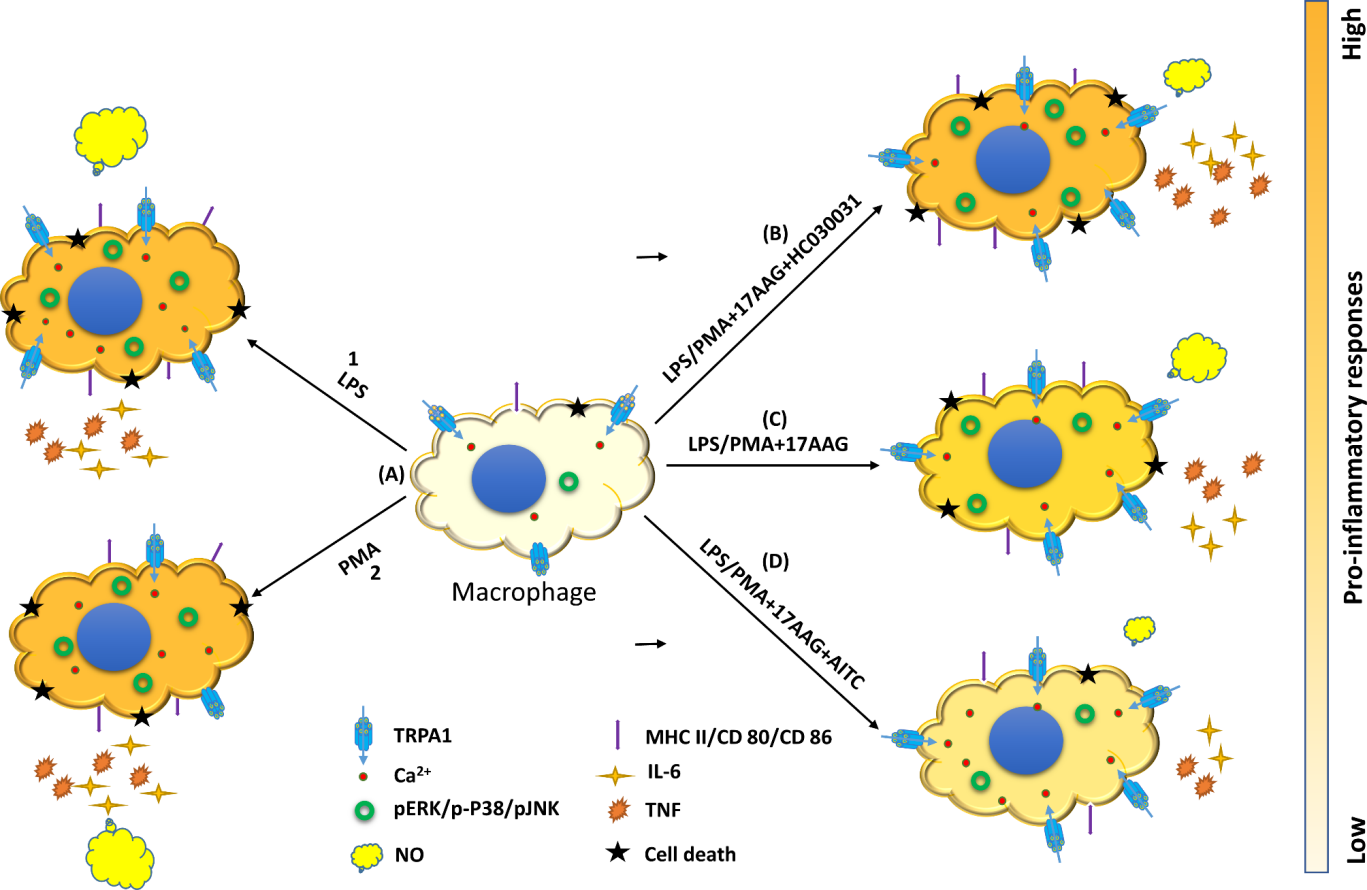
A proposed comprehensive working model. A detailed working model depicting the role of TRPA1 in 17-AAG mediated regulation of inflammation in LPS or PMA stimulated macrophages. TRPA1 is modulated upon LPS/PMA stimulation (**A**). pro-inflammatory responses including IL-6, TNF, MHCII, CD80/86, NO, intracellular calcium, and intracellular signaling proteins p38-MAPK, p-ERK 1/2, p-SAPK-JNK are significantly upregulated with LPS (**A.1**) or PMA stimulation (**A.2**). Upon administration of 17-AAG along with LPS or PMA, the pro-inflammatory responses are downregulated (**C**). Inhibition of TRPA1 via HC-030031 with 17-AAG and LPS or PMA administration reverses the pro-inflammatory responses, intracellular signaling proteins p38-MAPK, p-ERK 1/2, p-SAPK-JNK back to the LPS or PMA stimulated levels with a further diminished intracellular calcium level (**B**). Activation of TRPA1 via AITC and 17-AAG in LPS or PMA stimulated macrophages by impairing the proinflammatory responses, intracellular calcium, and intracellular signaling proteins p-p38-MAPK, p-ERK 1/2, p-SAPK-JNK to a greater extent exhibiting an anti-inflammatory property (**D**). The pro-inflammatory responses are represented according to the color code.

Together, these results suggest the anti-inflammatory nature of TRPA1 in LPS-stimulated macrophages and its synergistic role in Hsp90 inhibition-mediated pro-inflammatory responses in macrophages. A proposed comprehensive working model of the same is depicted in Fig. 8.

## Discussion

The role of TRPA1, a non-selective cation channel, and Hsp90, a chaperone molecule in various immune responses has been well studied over recent years. TRPA1 plays an essential role in many immune cells, including T cells, macrophages, and monocytes [11–15]. The potential of TRPA1 in regulating various inflammatory pathways and its association with various intracellular proteins has provided insights toward TRPA1 targeted therapeutic development in various autoimmune disorders and infectious diseases [12, 19, 20, 25, 26, 71, 72]. Similarly, Hsp90 has been well attributed as a critical component in regulating various immune responses. Inhibition of HSp90 via various biological and synthetic compounds is effective in downregulating various inflammatory responses, including monocyte/macrophage-associated pro-inflammatory responses [34–39]. Even though these two molecules are effective modulators of inflammatory responses, their possible associations or the functional regulation between them in inflammatory responses are not yet been assessed. Our study highlights the role of TRPA1 channels in regulating the anti-inflammatory effect of Hsp90 inhibition. We also emphasized a novel approach to downregulate macrophage-mediated pro-inflammatory responses. We have chosen mouse RAW 264.7, and human THP-1 macrophages stimulated with LPS or PMA as model systems for studying the pro-inflammatory responses. Hsp90 inhibition mediated downregulation of macrophage activation was obtained through 17-AAG.

This study suggests an important role of a TRP channel in regulating the Hsp90 inhibitor’s effect on inflammatory responses. Additionally, we have demonstrated the association of TRPA1 with Hsp90 in inflammatory responses in vitro. Our results suggest that TRPA1 has an anti-inflammatory role in 17-AAG-mediated development in macrophages during inflammation, supporting various other studies. TRPA1 is associated with and upregulated in various inflammatory conditions [14, 15, 73, 74, 17, 23, 51–55, 69]. Here, we have demonstrated that TRPA1 is upregulated during LPS/PMA stimulation and further augmented with 17-AAG administration in macrophages. Additionally, the frequency of TRPA1 positive cells and expression was significantly increased upon LPS stimulation, while it was diminished upon PMA stimulation. This might be a reflection of the different activation mechanisms these molecules induce. Although these expression patterns are previously reported, the actual mechanisms behind these observations are yet to be reported. Furthermore, we highlight that the effect of Hsp90 inhibition in TRPA1 positive cell frequency is augmented in a time-dependent and reversible manner. This elevation of TRPA1 in macrophages indicates a possible association of TRPA1 in 17-AAG-induced Hsp90 inhibition-mediated anti-inflammatory developments in LPS or PMA-stimulated macrophages.

Here in this report, we have examined the effect of TRPA1 in 17-AAG-mediated downregulation of various inflammatory responses in macrophages. This study addresses the essential role of a TRP channel in regulating the Hsp90 inhibitor’s effect on inflammatory responses. Additionally, we have demonstrated the association of TRPA1 with Hsp90 in inflammatory responses in vitro. Our results suggest that TRPA1 has an anti-inflammatory role in 17-AAG-mediated development in macrophages during inflammation, supporting various other studies. 17-AAG is widely reported to regulate various autoimmune disorders and inhibit the TLR4-mediated inflammatory signaling cascade in macrophages [47, 48]. Our findings support these studies as 17-AAG administration in macrophages stimulated with LPS/PMA has significantly downregulated the LPS-induced pro-inflammatory responses in macrophages such as cell surface expression of MHCII, CD80, CD86, production of NO and inflammatory cytokines (TNF and IL-6). Our work highlights the regulatory role of TRPA1 in this case. The results suggest that the pharmacological modulation of TRPA1 has a significant impact on the 17-AAG-mediated anti-inflammatory developments in LPS/PMA-stimulated macrophages. HC-030031-mediated inhibition of TRPA1 has significantly impaired the suppression of pro-inflammatory responses in LPS/PMA-stimulated macrophages. HC-030031 administration, along with 17-AAG and LPS, has abolished or diminished the 17-AAG-mediated downregulation of inflammatory responses as the MHCII, CD80, CD86 surface expression, inflammatory cytokines such as TNF and IL-6 production were comparable or higher than that of LPS/PMA stimulated macrophages, clearly depicting that TRPA1 is important for 17-AAG-mediated anti-inflammatory responses. Further, the TRPA1 activation via AITC has significantly augmented the 17-AAG-mediated downregulation of inflammation as MHCII, CD80, and CD86 surface expression, secretion of inflammatory cytokines such as TNF and IL-6, and NO production are the lowest. Hsp90 inhibition in macrophages follows a suppression of various macrophage-activation signaling processes. Macrophage activation via LPS or PMA induces apoptosis due to various secreted molecules such as NO and inflammatory cytokines. Our observations suggest that macrophages are prone to apoptosis at 12–24 h post-stimulation. Additionally, Hsp90 is well known to be involved in various cell fate decisions, including cell differentiation and apoptosis [35, 36, 39]. Our results suggest that TRPA1 has a significant regulatory role in the 17-AAG-mediated downregulation of apoptosis. Functional activation of TRPA1 could significantly increase cell death compared to the 17-AAG and LPS administration in macrophages. Similarly, the 17-AAG-mediated downregulation apoptosis was reversed by HC-030031-mediated TRPA1 inhibition.

Our experiments on various signaling protein expressions upon Hsp90 inhibition with TRPA1 modulators suggested that TRPA1 has a critical role in Hsp90 inhibition-mediated down-regulation of macrophage activation signaling. The MAPK signaling proteins p-p38-MAPK, p-ERK 1/2, and p-SAPK/JNK expressions were significantly downregulated with 17-AAG treatment in LPS-stimulated macrophages as expected. Interestingly, TRPA1 inhibition via HC-030031 has reversed the downregulated expression of signaling proteins p-p38-MAPK, p-ERK 1/2, p-SAPK/JNK by 17-AAG, indicating that TRPA1 is required for the 17-AAG-mediated downregulation of MAPK signaling pathways. Furthermore, the expression of the signaling proteins p-p38-MAPK, p-ERK 1/2, and p-SAPK/JNK were successfully diminished with TRPA1 activation and Hsp90 inhibition via 17-AAG. Modulation of the signaling cascade can be the active mechanism behind the effect of TRPA1 in regulating Hsp90 inhibition-mediated anti-inflammatory effects. These results suggest that TRPA1 and its activation may augment the efficiency of 17-AAG in ant-inflammatory responses, leading to a novel combinatorial approach to regulating inflammatory responses.

Even though TRP channels are considered non-selective cation channels, the calcium influx through these channels has an important role in TRP channel functions. A high Ca^2+^ influx is preceded by LPS stimulation in macrophages. Our results demonstrated that 17-AAG (Hsp90 antagonist) or HC-030031 (TRPA1 antagonist) administration significantly diminishes LPS-mediated elevation of intracellular calcium in macrophages. The TRPA1 activation has augmented these Ca^2+^ levels, while the TRPA1 antagonist has further reduced it, indicating that the calcium influx occurring via LPS and 17-AAG administration is dependent on TRPA1. These changes in Ca^2+^ levels could reflect the activity of additional TRPA1 channels recruited by 17-AAG administration in LPS-stimulated macrophages. This Ca^2+^ status may not correspond to calcium levels for the regulatory role of TRPA1 in the 17-AAG (Hsp90 antagonist)-mediated effect. Still, it may trigger various downstream signaling cascades that lead to the observed anti-inflammatory developments by 17-AAG.

The present study may have implications for the synergistic role of TRPA1 activation and Hsp90 inhibition toward developing future regulatory measures against various inflammatory responses. The future perspective of the study may include the sub-cellular mechanism associated with domains of TRPA1 and 17-AAG interactions and the replication of these results in different inflammatory models.

## Conclusion

In conclusion, our study indicates an important role of TRPA1 in Hsp90 inhibition-mediated anti-inflammatory developments in LPS or PMA-stimulated macrophages. TRPA1 activation and Hsp90 inhibition synergistically may regulate the inflammatory responses in macrophages and this combinatorial approach may have implications towards designing future therapeutic strategies in various diseases and inflammatory disorders.

## Materials and methods

### Cell culture

Mouse macrophage cell line, RAW 264.7 (source – ATCC (ATCC® TIB-71™)) was cultured in complete Roswell Park Memorial Institute-1640 medium (RPMI-1640) (PAN Biotech, Aidenbach, Germany) with penicillin (100 U/mL), Streptomycin (0.1 mg/mL), and 2.0 mM L-Glutamine (Himedia Laboratories Pvt. Ltd., Mumbai, MH, India), 10% heat-inactivated fetal bovine serum (FBS) (PAN Biotech, Aidenbach, Germany) at 37ºC in a sterile incubator with 5% CO_2_ and appropriate humidity. Enzyme-free cell dissociation reagent (ZymeFree™; Himedia Laboratories Pvt. Ltd, Mumbai, MH, India) was used to maintain the cells [56].

Undifferentiated human leukemia monocytic cell line, THP-1 (source – ATCC (ATCC® TIB-202™)) was maintained in complete RPMI-1640 (PAN Biotech, Aidenbach, Germany) supplemented with Penicillin (100 U/mL), Streptomycin (0.1 mg/mL), and 2.0 mM L-Glutamine (Himedia Laboratories Pvt. Ltd., Mumbai, MH, India), 10% heat-inactivated FBS (PAN Biotech, Aidenbach, Germany) at 37ºC in a sterile incubator with 5% CO_2_ and appropriate humidity. THP-1 cells were further treated with 100 ng/ml PMA for 24 h to differentiate monocytes into macrophage-like cells [75].

### Antibodies, reagents, and pharmacological modulators

Rabbit polyclonal antibody against extracellular TRPA1 with specific blocking peptide [TRPA1, INSTGIINETSDHSE] was obtained from Alomone Laboratories (Jerusalem, Israel). Mouse antibodies against Hsp90, CD80, CD86, I-Ad/I-Ed (MHCII) were purchased from BD Biosciences (SJ, USA). The anti-mouse Alexa Fluor 647 (AF-647), anti-rabbit Alexa Fluor 488 (AF-488), and Fluo-4 AM were procured from Invitrogen (Carlsbad, CA, USA). Mouse IgG1 isotype control and rabbit IgG1 isotype control were bought from Abgenex India Pvt. Ltd. (Bhubaneswar, India). Saponin and Bovine serum albumin (BSA) fraction-V were procured from Merck Millipore (Billerica, MA, USA). 17-AAG and the pharmacological modulators of TRPA1 channel-antagonist HC-030031, agonist Allyl isothiocyanate (AITC) were purchased from Alomone Laboratories (Jerusalem, Israel). HC-030031 and AITC are proven to be functional modulators of TRPA1, and their administration may not alter the TRPA1 expression levels.

### Cell viability assay

To assess the cytotoxicity of pharmacological modulators, RAW 264.7 and PMA differentiated THP-1 macrophages were administrated with differential doses of TRPA1 modulators HC-030031 and AITC along with 17-AAG (0.5 µg/ml) and incubated for 24 h. Cells were immediately assessed by trypan blue exclusion assay. Additionally, samples were stained with Annexin V and 7-AAD and evaluated for cell viability. The percentage of viable cells was calculated in comparison to the control cells.

### LPS/PMA stimulation in macrophages

RAW 264.7 cells were harvested and seeded in a six-well plate. After the cells had reached monolayer confluency of ~ 80%, they were washed with 1X PBS and subjected to LPS (500 ng/ml) or PMA (100 ng/ml) [76] dissolved in fresh complete RPMI-1640. The supernatant and the cells were then harvested. They were stored or processed at various time points. 17-AAG was used to promote the Hsp90-inhibited condition in the cells. For assessing the effect of TRPA1 in the Hsp90-inhibited cells in presence of LPS-induced inflammatory responses, RAW 264.7 cells were subjected to 17-AAG (0.5 µM) and TRPA1 pharmacological modulators incubation followed by LPS or PMA administration for 6 to 24 h. DMSO was used as solvent control. The further proceedings were executed by following the protocols mentioned above.

Similarly, undifferentiated THP-1 cells were harvested and seeded in a six-well plate with the administration of 100 ng/ml PMA for 24 h. After the cells reached a monolayer confluency of ~ 80%, they were washed with 1X PBS and kept in PMA-free media for another 24 h before LPS treatment (500 ng/ml) [77]. PMA-differentiated THP-1 macrophages were stimulated with LPS only for later experiments and no further PMA stimulation was carried out. Post-treatment, the supernatant, and the cells were harvested, then stored or processed at various time points. Further experiments were conducted as mentioned above.

### Indirect immunofluorescence and Flow cytometry (FC)

RAW 264.7 and THP-1 macrophages were subjected to different stimuli and pharmacological modulators of TRPA1 and Hsp90. For TRPA1 extracellular staining, cells were harvested, resuspended in staining buffer (1X PBS, 1% BSA, 0.01% NaN_3_), and then incubated with primary anti-mouse TRPA1 antibody for 30 min on ice. After direct staining, any excess unbound antibody was washed out with an additional staining buffer. Subsequently, a secondary fluorochrome-conjugated (AF-488) antibody was administrated and incubated for 30 min followed by washing with staining buffer. The rabbit IgG was used as isotype control [56]. Samples (10,000 cells/sample) were then acquired via BD FACS Calibur/BD LSRFortessa (BD Biosciences) and analyzed using FlowJo v10.8.1.

Cell death/apoptosis analysis was conducted using PE Annexin V Apoptosis Detection Kit I (BD Biosciences). Freshly harvested cells were washed with 1X PBS followed by incubation with Annexin V in Annexin V binding buffer for 15 min. The assay was conducted by following the manufacturer’s protocol [78]. Samples were immediately acquired via FC and analyzed using FlowJo v10.8.1.

### Enzyme-linked Immunosorbent Assay (ELISA)

ELISA was performed to quantify and analyze the cytokine levels in RAW 264.7 and THP-1 cell culture supernatants under different experimental conditions of LPS/PMA,17-AAG, HC-0300031, and AITC. Sandwich ELISA was executed using the BD OptEIA™ sandwich ELISA kit (BD Biosciences) following the instructions of the manufacturer’s protocol [56]. The cytokine concentration in each sample was estimated in pg/ml from the standard curve.

### Western blotting

To analyze the expression of various proteins of interest, a western blot was performed after stimulating the cells with LPS (500 ng/ml) and pharmacological modulators. In brief, respective cells (RAW 264.7 and THP-1) were treated with LPS (500 ng/ml) for 15 min, washed with 1X PBS, and immediately harvested. Cell lysis, protein estimation, and western blotting were done according to the protocol mentioned earlier [56]. Briefly, cells were harvested and washed with 1X PBS and whole cell lysates (WCL) were prepared using Radio Immuno Precipitation Assay (RIPA) buffer. The lysates were centrifuged at 13,000 rpm for 30 min at 4 °C. The protein concentrations were then quantified using the Bradford reagent (Sigma-Aldrich). The same amount of proteins was loaded in 10% SDS-gel. After running, the gels were blotted on a PVDF membrane (Millipore, MA, USA) and then blocked by 3% BSA in TBST. The blots were cut before antibody staining, then incubated overnight with primary antibodies, Hsp90 (1:1000), TRPA1 (1:1000), GAPDH (1:5000), Cleaved Caspase 3 (1:1000), p38 (1:2000), SAPK/JNK (1:2000), ERK (1:2000). The blots were then washed with TBST, 3 times, 5 min each. Then, HRP-conjugated secondary antibodies were added and blots were incubated for 2 h at RT. Blots were then washed with TBST, 3 times, 5 min each, and chemiluminescent detection reagent (Immobilon Western Chemiluminescent HRP substrate, Millipore) was added and images were captured by ChemiDoc (Bio-Rad). The ImageLab analysis software was used for band intensity quantification of western blot images with normalization to the corresponding loading controls.

### Calcium (Ca^2+^) influx analysis

To evaluate the Ca^2+^ influx after the administration of various stimuli and pharmacological modulators, the cells were incubated with 5 µM Fluo-4 AM for 45–60 min in HBSS buffer and subjected to de-esterification for 15 min in 1X PBS. Cells were then incubated in an HBSS medium and Ca^2+^ influx was analyzed in FC by measuring the time-dependent fluorescence intensity upon adding the respective modulators, as mentioned earlier [79]. The data were analyzed using FlowJo for kinetic studies and obtaining mean fluorescence every 10 s.

### Nitrite estimation

The supernatant of macrophages was treated with the differential conditions of LPS/PMA,17-AAG, HC-0300031, and AITC for 6, 12, and 24 h in colorless RPMI-1640. About 100 µl of supernatants were used to measure NO levels with 100 µl of 1% sulfanilamide and 100 µl of 0.1% N-1-naphthylenediamine dihydrochloride [80]. Samples were incubated for 10 min and the absorbance values were read at 540 nm using a microplate reader (Epoch 2 microplate reader, BioTek, USA). Nitrite concentrations were calculated from a standard graph prepared using different concentrations of sodium nitrite dissolved in colorless RPMI-1640.

### Statistical analysis

Statistical analyses were performed using GraphPad Prism 9.0 software (GraphPad Software Inc., San Diego, CA, USA). The comparison between the groups was performed by one-way ANOVA or two-way ANOVA with the Bonferroni posthoc test unless otherwise mentioned. The data is represented as the mean ± SD of three independent experiments (n = 3). p < 0.05 was reflected as a statistically significant relation between the respective groups (ns, non-significant; * *p* < 0.05; ** *p* < 0.01; *** *p* < 0.001).

## Electronic supplementary material

Below is the link to the electronic supplementary material.

Additional File 1: Supplementary/Supporting Informations

## Data Availability

The datasets generated during and/or analyzed during the current study are available from the corresponding author upon reasonable request.

## List of Abbreviations

TRPA1: Transient receptor potential ankyrin channel 1
Hsp90: Heat shock protein 90
17-AAG: 17-(allylamino)-17-demethoxygeldanamycin
LPS: Lipopolysaccharide
PMA: Phorbol 12-myristate 13-acetate
AITC: Allyl isothiocyanate
HC-030031: 1,2,3,6-Tetrahydro-1,3-dimethyl-N-[4-(1-methylethyl) phenyl]-2,6-dioxo-7 H-purine-7- acetamide,2-(1,3-Dimethyl-2,6-dioxo-1,2,3,6-tetrahydro-7 H-purin-7-yl)-N-(4-isopropylphenyl) acetamide
MHCII: Major histocompatibility complex II
CD80: Cluster of differentiation 80
CD86: Cluster of differentiation 86
TNF: Tumor necrosis factor
IL-6: Interleukin 6
NO: Nitric oxide
MAPK: Mitogen-activated protein kinase
ERK: Extracellular signal-regulated kinase
SAPK: Stress-Activated Protein Kinase
JNK: C-Jun N-terminal kinase
Lck: Lymphocyte-specific protein tyrosine kinase
FBS: Fetal bovine serum
FC: Flow cytometry
DMSO: Dimethyl sulfoxide
