# Supplementary material for: TRPA1 activation and Hsp90 inhibition synergistically downregulate macrophage activation and inflammatory responses in vitro
Source: BMC Immunol. 2023 Jun 30;24:16. doi: 10.1186/s12865-023-00549-0 (PMC10314470; doi:10.1186/s12865-023-00549-0)
Supplement: Supplementary file 1 — Additional File 1: Supplementary/Supporting Informations [file 12865_2023_549_MOESM1_ESM.pdf]

TRPA1 activation and Hsp90 inhibition synergistically downregulate macrophage activation and inflammatory responses *in vitro*.

Anukrishna Radhakrishnan[a#], Tathagata Mukherjee[a#], Chandan Mahish[a#], P Sanjai Kumar[b], Chandan Goswami[a], Subhasis Chattopadhyay[a†]

a National Institute of Science Education and Research, an OCC of Homi Bhabha National Institute, Bhubaneswar 752050, Odisha, India. anukrishnaradhakrishnan@gmail.com, tathagatam007@gmail.com, chandan1990.13@gmail.com, chandan@niser.ac.in, subho@niser.ac.in[†]

b Institute of Life Sciences, Nalco Nagar Rd, NALCO Square, NALCO Nagar, Chandrasekharpur, Bhubaneswar, Odisha 751023, India. sanjai27112009@gmail.com

# Equal contributions

† Corresponding author

Supplementary information

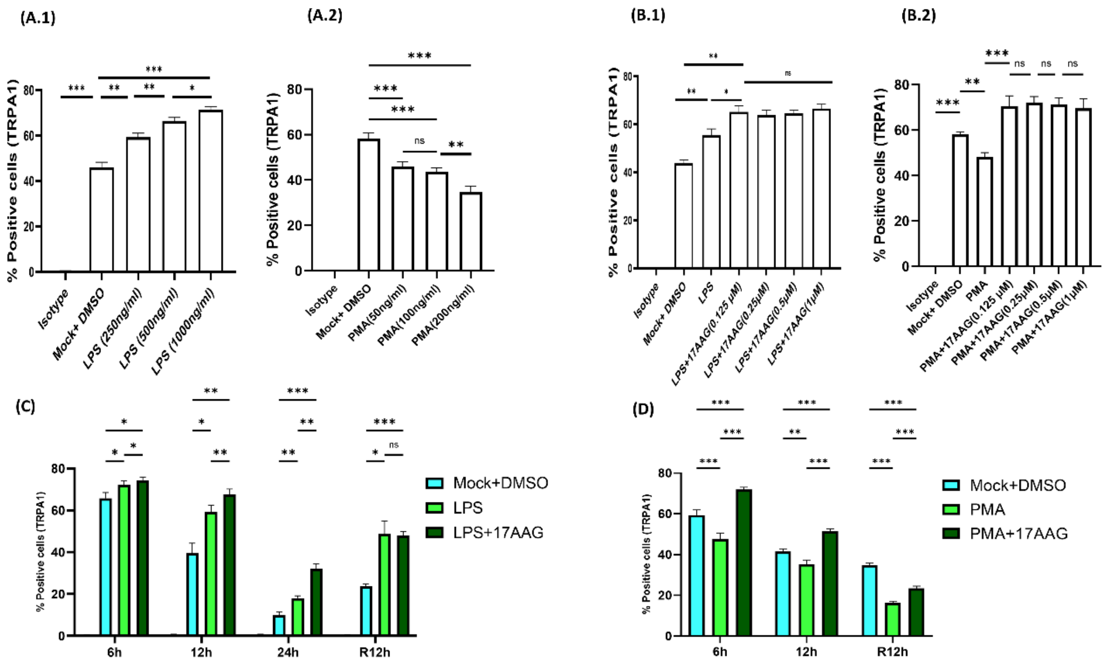

Supplementary fig.1 Dose and time kinetics of TRPA1 expression in LPS/PMA-stimulated and Hsp90-inhibited macrophages. RAW 264.7 cells were treated with different doses of LPS, PMA, and 17-AAG for 12 h. (A) Bar graph representing the percentage of positive cells for TRPA1 with different doses of LPS (A.1) / PMA (A.2). (B) Bar graph representing the percentage of positive cells for TRPA1 for differential doses of 17-AAG + LPS (500 ng) (B.1) / PMA (100 ng) (B.2). (C) Bar graph comparing the percentage of positive cells for TRPA1 in the presence of LPS or LPS + 17AAG condition or (D) PMA, PMA + 17-AAG at different time points. R represents time points after the reversal of conditions (replacing the existing culture media with fresh media containing only DMSO). The data represent the mean ± SD of three independent experiments. One-way ANOVA was used for A and B, and two-way ANOVA was used for C. Differences between groups with a p-value less than 0.05 were considered statistically significant (ns, non-significant; *, $p < 0.05$; **, $p < 0.01$; ***, $p < 0.001$).

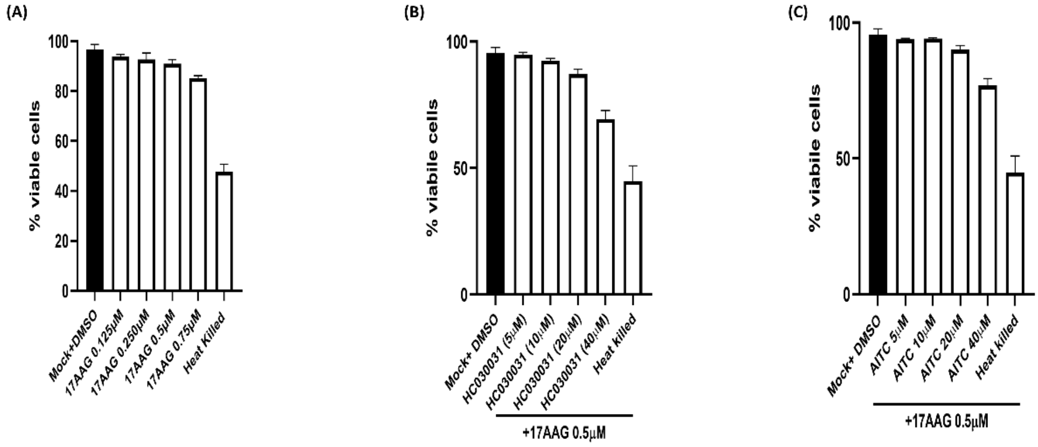

Supplementary fig.2 Cell viability assay for TRPA1 modulators (AITC and HC-030031) in Hsp90-inhibited macrophages. RAW 264.7 cells were treated with different doses of 17-AAG or AITC and HC-030031 in the presence of 0.5 µM of 17-AAG. Cells were harvested at 24 h and immediately stained with 7-AAD and analyzed via FC. (A) Bar graph showing the percentage of viable RAW 264.7 cells at different doses of 17-AAG. (B) Bar graph representing the percentage of viable RAW 264.7 cells at different doses of AITC (B) and (C) HC-030031 in Hsp90-inhibited macrophages. The data represent the mean ± SD of three independent experiments. One-way ANOVA has been performed for statistical significance analysis. Differences between groups with a p-value < 0.05 were considered statistically significant (***, $p < 0.001$).

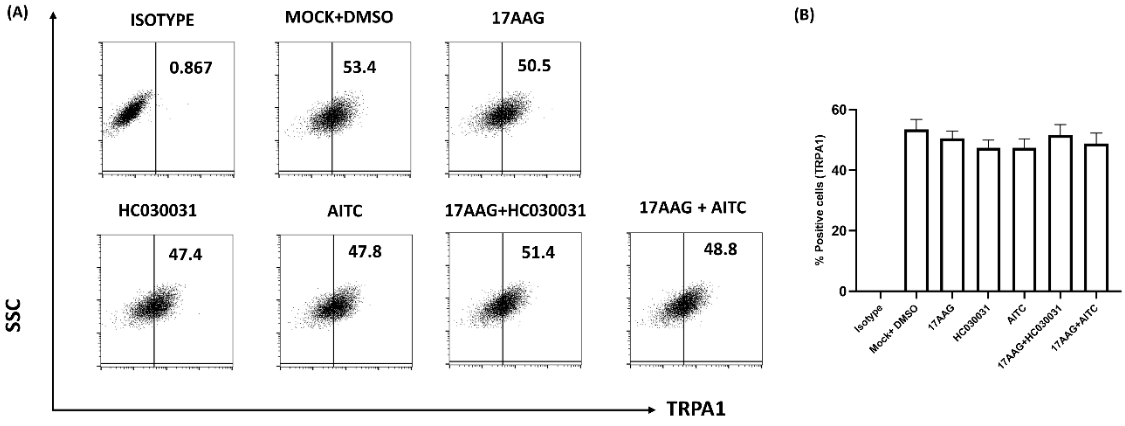

Supplementary fig.3 Expression levels of TRPA1 in the presence of TRPA1 modulators alone or in combination with 17-AAG in RAW 264.7 cells. Supplementary fig.3 Expression levels of TRPA1 in the presence of TRPA1 modulators alone or in combination with 17-AAG in RAW 264.7 cells. (A) FC dot-plot depicting the percentage of positive cells for TRPA1 in differential concentrations of AITC and HC-030031 alone or with 17-AAG and (B)representative bar graph showing the same. The data represent the mean ± SD of three independent experiments. One-way ANOVA has been performed to find statistical significance among groups. Differences between groups with a p-value < 0.05 were considered statistically significant.

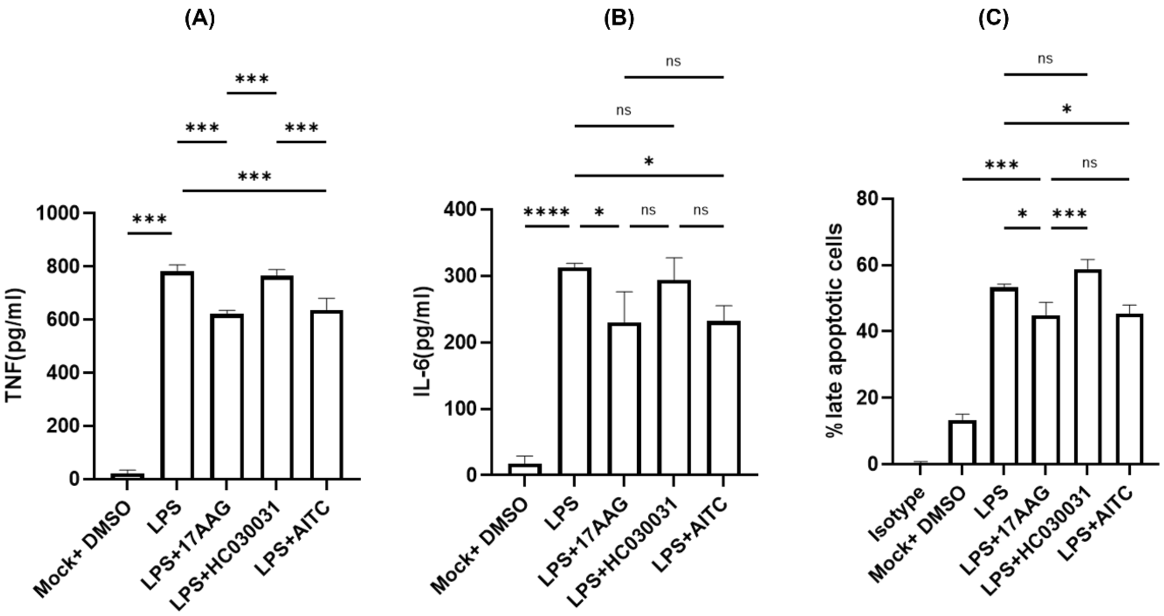

Supplementary fig.4 Effect of TRPA1 modulators in the absence of Hsp90 inhibitor in regulating LPSinduced inflammation. Bar graph representing the quantity of TNF (A) and IL-6 (B) production in RAW 264.7 cells under differential conditions of TRPA1 modulator and LPS at 12 h. (C) bar graph representing the % of late apoptotic cells from RAW 264.7 cells under differential conditions of TRPA1 modulator and LPS at 24 h. The data represent the mean ± SD of three independent experiments. One-way ANOVA has been performed to find statistical significance among groups Differences between groups with a p-value less than 0.05 were considered statistically significant (ns, non-significant; *, $p < 0.05$; **, $p < 0.01$; ***, $p < 0.001$).

Western blot RAW images:

- **Figure 1 of the manuscript:**

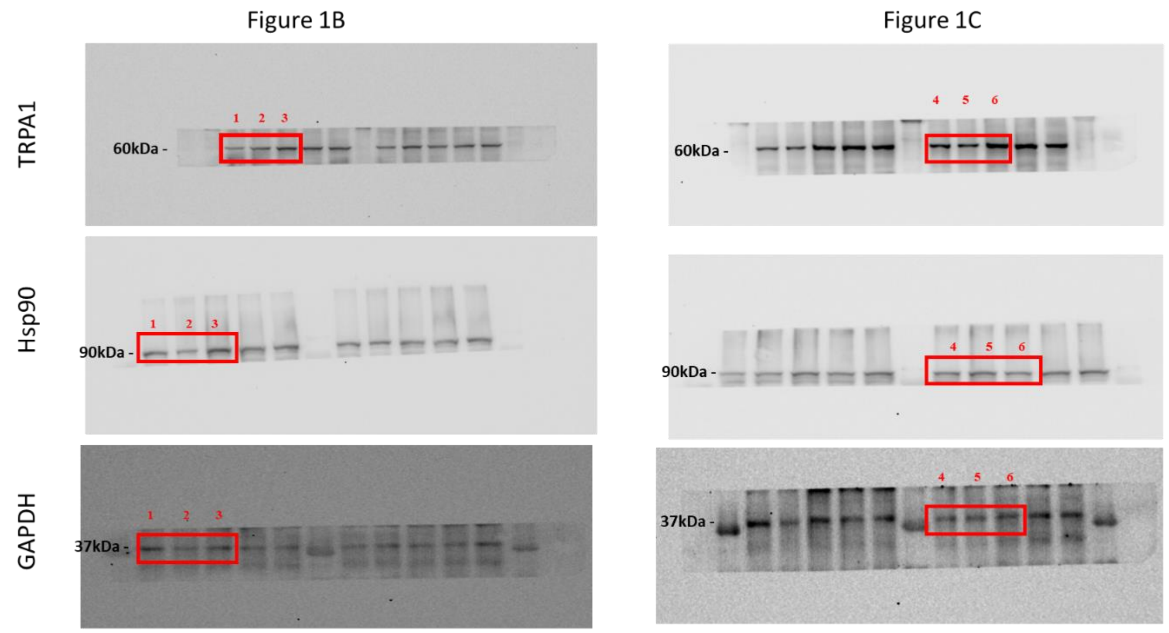

Lanes:
1 – Mock, 2 – LPS, 3 – LPS+ 17-AAG, 4 –Mock, 5 –PMA, 6 – PMA+ 17-AAG

Fig: **Raw images for Figures 1 B and 1 C**.

- **TRPA1 Replicates:**

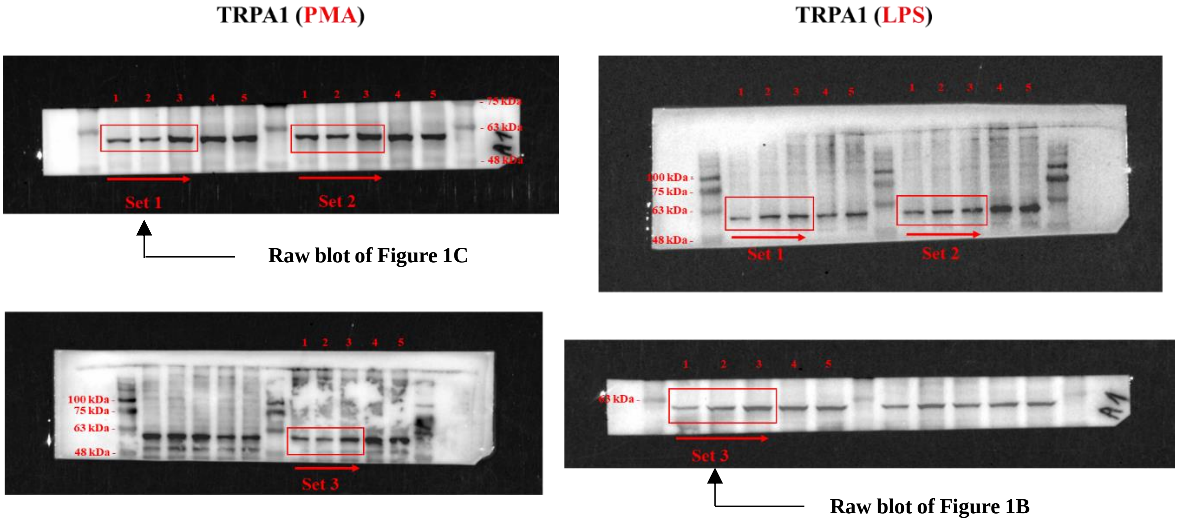

Lanes:
1 – Mock, 2 – LPS/PMA, 3 – LPS/PMA + 17-AAG, 4 – LPS/PMA + 17-AAG + HC, 5 – LPS/PMA + 17-AAG + AITC

Fig: **Raw images of TRPA1 replicates**.

➢ Hsp90 Replicates:

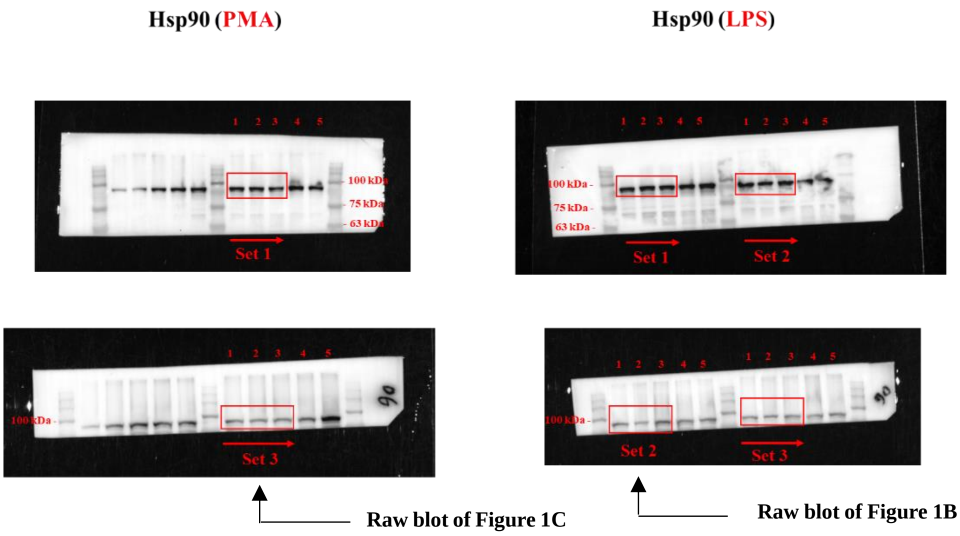

Lanes:
1 – Mock, 2 – LPS/PMA, 3 – LPS/PMA + 17-AAG, 4 – LPS/PMA + 17-AAG + HC, 5 – LPS/PMA + 17-AAG + AITC

Fig: **Raw images of Hsp90 replicates**.

➢ Figure 6 of the manuscript:

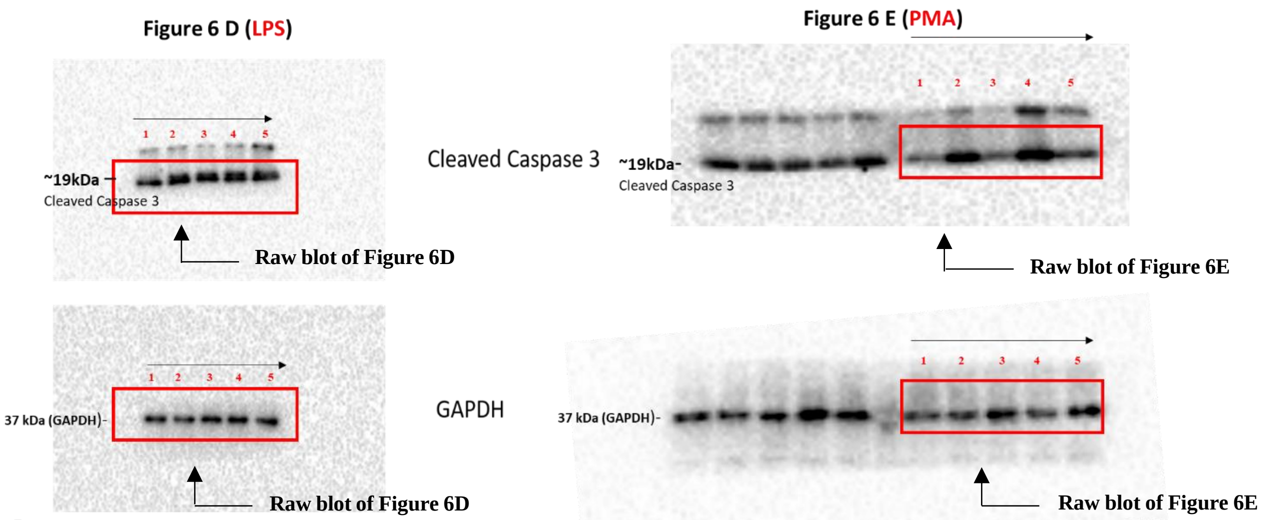

Lanes:
1 – Mock, 2 – LPS/PMA, 3 – LPS/PMA + 17-AAG, 4 – LPS/PMA + 17-AAG + HC, 5 – LPS/PMA + 17-AAG + AITC

Fig: **Raw images of Figures 6 D and 6E**.

➢ Cleaved Caspase 3 Replicates:

Cleaved caspase 3 (PMA)

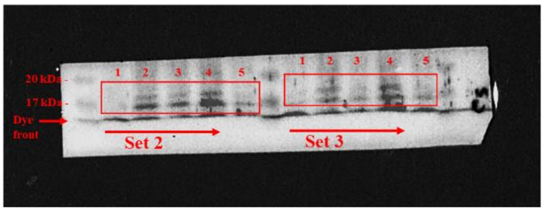

Cleaved caspase 3 (LPS)

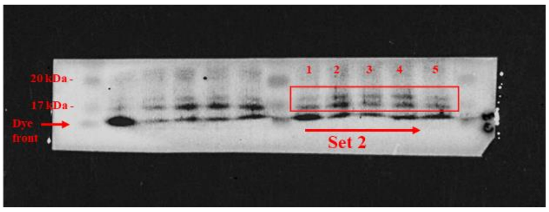

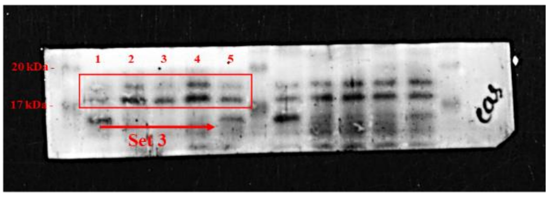

Lanes:
1 – Mock, 2 – LPS/PMA, 3 – LPS/PMA + 17-AAG, 4 – LPS/PMA + 17-AAG + HC, 5 – LPS/PMA + 17-AAG + AITC

Fig: **Raw images of cleaved caspase 3 replicates**.

➢ GAPDH Replicates:

GAPDH (PMA)

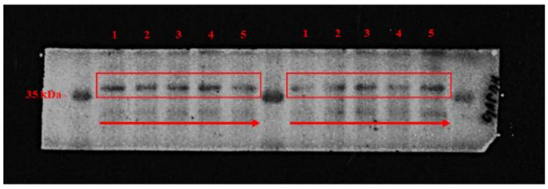

GAPDH (LPS)

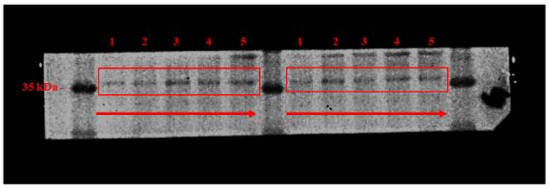

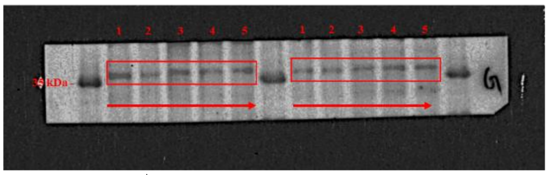

Raw blot of Figure 1C

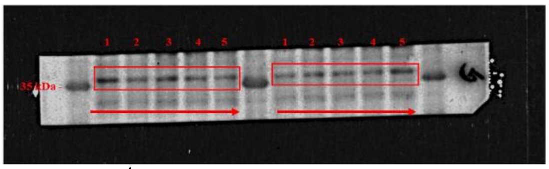

Raw blot of Figure 1B

Lanes:
1 – Mock, 2 – LPS/PMA, 3 – LPS/PMA + 17-AAG, 4 – LPS/PMA + 17-AAG + HC, 5 – LPS/PMA + 17-AAG + AITC

Fig: **Raw images of GAPDH replicates**.

➢ Figure 5 of the manuscript:

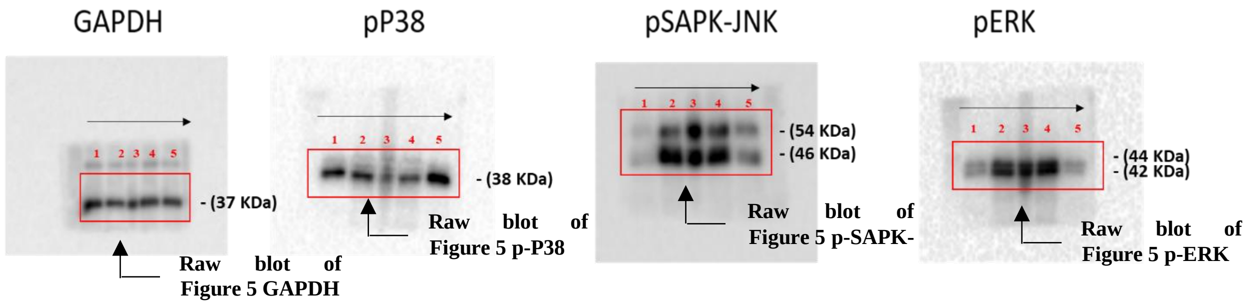

Lanes:
1 – Mock, 2 – LPS/PMA, 3 – LPS + 17-AAG, 4 – LPS + 17-AAG + HC, 5 – LPS + 17-AAG + AITC

Fig: **Raw images of p-P38, p-SAPK-JNK and p-ERK**.

➢ p-P38 Replicates:

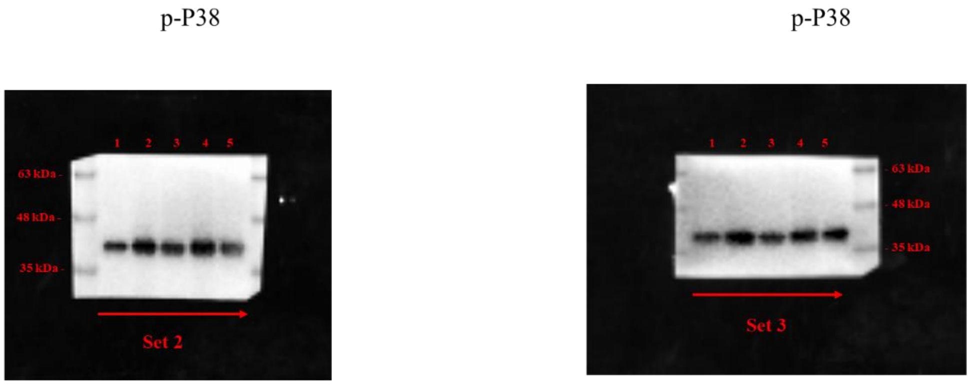

Lanes:
1 – Mock, 2 – LPS, 3 – LPS + 17-AAG, 4 – LPS + 17-AAG + HC, 5 – LPS + 17-AAG + AITC

Fig: **Raw images of p-P38 replicates**.

➢ p-SAPK-JNK Replicates:

p-SAPK-JNK

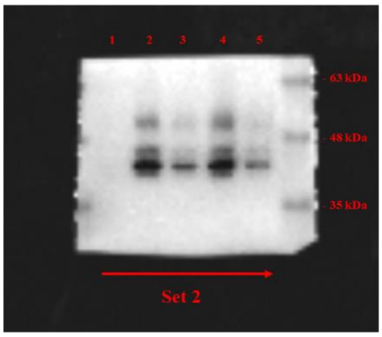

p-SAPK-JNK

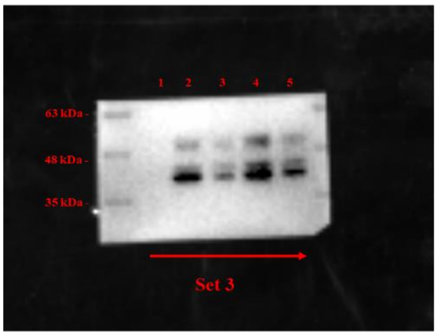

Lanes:
1 – Mock, 2 – LPS, 3 – LPS + 17-AAG, 4 – LPS + 17-AAG + HC, 5 – LPS + 17-AAG + AITC

Fig: **Raw images of p-SAPK-JNK replicates**.

➢ p-ERK Replicates:

p-ERK 1/2

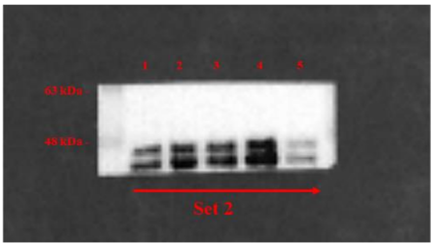

p-ERK 1/2

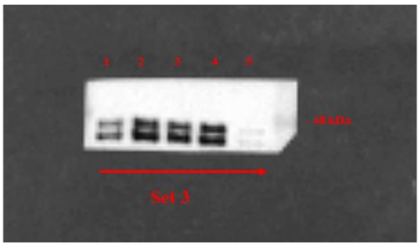

Lanes:
1 – Mock, 2 – LPS, 3 – LPS + 17-AAG, 4 – LPS + 17-AAG + HC, 5 – LPS + 17-AAG + AITC

Fig: **Raw images of p-ERK replicates**.

➢ GAPDH Replicates:

GAPDH GAPDH

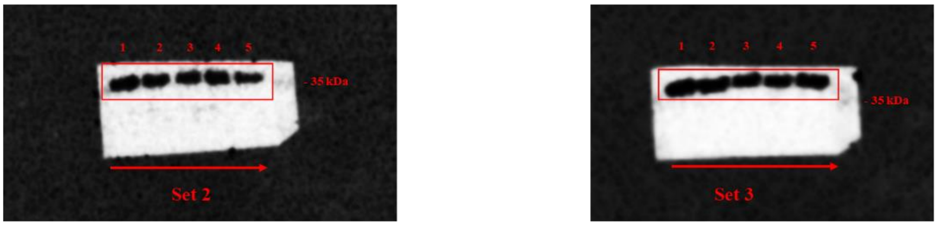

Lanes:
1 – Mock, 2 – LPS, 3 – LPS + 17-AAG, 4 – LPS + 17-AAG + HC, 5 – LPS + 17-AAG + AITC

Fig: **Raw images of GAPDH replicates**.

➢ Table S1: Change in Fluo-4 AM intensity in RAW 264.7 cells with time in presence of different experimental treatments.

Time(s)	Mock	LPS	Ionomycin	LPS+17AAG	LPS+17AAG+HC030031	LPS+17AAG+AITC	LPS+HC030031	LPS+AITC
	Mean ± standard deviation							
0-20	1.00 ± 0.000	1.00 ± 0.00	1.00 ± 0.00	1.00 ± 0.00	1.00 ± 0.00	1.00 ± 0.00	1.00 0.00	1.00 0.00
20-40	0.98 ± 0.020	2.38 ± 0.58	1.41 ± 0.02	0.94 ± 0.12	0.91 ± 0.12	1.09 ± 0.12	1.03 0.07	1.59 0.15
40-60	0.98 ± 0.052	1.73 ± 0.24	1.58 ± 0.07	0.98 ± 0.07	0.90 ± 0.10	1.15 ± 0.17	1.11 ± 0.12	1.59 ± 0.12
60-80	0.94 ± 0.040	1.37 ± 0.38	1.44 ± 0.04	0.95 ± 0.06	0.83 ± 0.06	1.22 ± 0.07	1.09 ± 0.12	1.48 ± 0.10
80-100	0.97 ± 0.043	1.27 ± 0.37	1.38 ± 0.03	0.95 ± 0.06	0.84 ± 0.07	1.26 ± 0.08	1.12 ± 0.12	1.43 ± 0.19
100-120	0.98 ± 0.020	1.25 ± 0.40	1.21 ± 0.14	0.95 ± 0.03	0.82 ± 0.03	1.30 ± 0.11	1.09 ± 0.13	1.32 ± 0.25
120-140	0.97 ± 0.035	1.24 ± 0.32	1.22 ± 0.21	0.96 ± 0.07	0.84 ± 0.07	1.29 ± 0.10	1.12 ± 0.18	1.14 ± 0.11
140-160	0.92 ± 0.058	1.31 ± 0.25	1.21 ± 0.20	0.95 ± 0.04	0.83 ± 0.05	1.29 ± 0.10	1.12 ± 0.16	1.24 ± 0.01
160-180	0.95 ± 0.025	1.14 ± 0.15	1.26 ± 0.05	0.97 ± 0.02	0.90 ± 0.04	1.30 ± 0.11	1.13 ± 0.21	1.35 ± 0.18
180-200	0.95 ± 0.020	1.07 ± 0.11	1.21 ± 0.02	1.00 ± 0.02	0.85 ± 0.03	1.34 ± 0.10	1.10 ± 0.15	1.14 ± 0.11

Table S1: **Fluo-4 AM MFI values obtained from RAW 264.7 cells under each experimental conditions and time interval**
